# Metagenomics Reveals Bacterial and Archaeal Adaptation to Urban Land-Use: N Catabolism, Methanogenesis, and Nutrient Acquisition

**DOI:** 10.3389/fmicb.2019.02330

**Published:** 2019-10-10

**Authors:** Dietrich J. Epp Schmidt, David Johan Kotze, Erzsébet Hornung, Heikki Setälä, Ian Yesilonis, Katalin Szlavecz, Miklós Dombos, Richard Pouyat, Sarel Cilliers, Zsolt Tóth, Stephanie Yarwood

**Affiliations:** ^1^Department of Environmental Science and Technology, University of Maryland, College Park, College Park, MD, United States; ^2^Ecosystems and Environment Research Programme, Faculty of Biological and Environmental Sciences, University of Helsinki, Lahti, Finland; ^3^Department of Ecology, University of Veterinary Science, Budapest, Hungary; ^4^Baltimore Ecosystem Study, USDA Forest Service, Baltimore, MD, United States; ^5^Department of Earth and Planetary Sciences, Johns Hopkins University, Baltimore, MD, United States; ^6^Institute for Soil Sciences and Agricultural Chemistry, Centre for Agricultural Research, Hungarian Academy of Sciences, Budapest, Hungary; ^7^Northern Research Station, University of Delaware, Newark, DE, United States; ^8^Unit for Environmental Sciences and Management, North-West University, Potchefstroom, South Africa

**Keywords:** urban, soil metagenomics, Ni-Fe hydrogenase, nitrification, microbiology, methanogenesis, DNRA, ammonia oxidation

## Abstract

Urbanization results in the systemic conversion of land-use, driving habitat and biodiversity loss. The “urban convergence hypothesis” posits that urbanization represents a merging of habitat characteristics, in turn driving physiological and functional responses within the biotic community. To test this hypothesis, we sampled five cities (Baltimore, MD, United States; Helsinki and Lahti, Finland; Budapest, Hungary; Potchefstroom, South Africa) across four different biomes. Within each city, we sampled four land-use categories that represented a gradient of increasing disturbance and management (from least intervention to highest disturbance: reference, remnant, turf/lawn, and ruderal). Previously, we used amplicon sequencing that targeted bacteria/archaea (16S rRNA) and fungi (ITS) and reported convergence in the archaeal community. Here, we applied shotgun metagenomic sequencing and QPCR of functional genes to the same soil DNA extracts to test convergence in microbial function. Our results suggest that urban land-use drives changes in gene abundance related to both the soil N and C metabolism. Our updated analysis found taxonomic convergence in both the archaeal and bacterial community (16S amplicon data). Convergence of the archaea was driven by increased abundance of ammonia oxidizing archaea and genes for ammonia oxidation (QPCR and shotgun metagenomics). The proliferation of ammonia-oxidizers under turf and ruderal land-use likely also contributes to the previously documented convergence of soil mineral N pools. We also found a higher relative abundance of methanogens (amplicon sequencing), a higher relative abundance of gene sequences putatively identified as Ni-Fe hydrogenase and nickel uptake (shotgun metagenomics) under urban land-use; and a convergence of gene sequences putatively identified as contributing to the nickel transport function under urban turf sites. High levels of disturbance lead to a higher relative abundance of gene sequences putatively identified as multiple antibiotic resistance protein marA and multidrug efflux pump mexD, but did not lead to an overall convergence in antibiotic resistance gene sequences.

## Introduction

In 2018, 55% of all humans reside in urban areas, and this figure is projected to increase to 68% by 2050 (United Nations [UN], 2018). Urban centers are a cultural nexus that determine the allocation of resources at a global scale (Friis et al., 2016) and alter global biogeochemical cycles (IPCC, 2007). They also exert top-down control of local ecosystems, resulting in a convergence (decreased variance globally across cities) of abiotic environmental parameters (Groffman et al., 2014) and the abundance and composition of plant, animal (McKinney, 2006) and microbial communities (Epp Schmidt et al., 2017). Biotic homogenization, a term often applied to urban community ecology to describe the convergence of community characteristics, may be driven by facilitated dispersion of invasive organisms or the extirpation of endemic species (McKinney and Lockwood, 1999). These mechanisms are intrinsic to the process by which humans are driving the next great extinction event (Baiser et al., 2012; Ceballos et al., 2017). We recently demonstrated that these mechanisms also drive convergence in urban soil microbial communities and may contribute to the reduction in ectomycorrhizal fungal diversity (Epp Schmidt et al., 2017).

In addition to biotic homogenization, urban ecosystem convergence includes soil physico-chemical parameters: soil pH, organic matter, and inorganic nitrogen (N) concentrations (Pouyat et al., 2015). For example, the widespread use of concrete buffers urban watersheds toward neutral pH, creating a unique lithology that when weathered releases calcium carbonate (Kaushal et al., 2013, 2014). Under ideal growing conditions, urban turfgrass sometimes sequesters as much as 500 g CO_2_/m^2^ annually (Townsend-Small and Czimczik, 2010); yet disturbed soils in urban environments often have lower C content (Pouyat et al., 2015). Industrial processes, including fossil fuel burning, lead to an enrichment of volatile N oxides and result in a higher rate of atmospheric N deposition and nutrient enrichment around urban centers (Galloway et al., 1994; Pouyat et al., 1995; Fenn et al., 2003; Juknys et al., 2007; Decina et al., 2017). Urban forests and riparian zones have higher N mineralization rates and net nitrification rates compared to reference ecosystems (Steinberg et al., 1997; Zhu and Carreiro, 1999; Zhu et al., 2006; Reisinger et al., 2016). Across large geographic scales, soil pH, organic matter, and total N are strongly related to soil microbial community structure (Fierer and Jackson, 2006; Fierer et al., 2009), suggesting that they are important in structuring microbial functional adaptation.

Due to an array of potential contaminating sources such as fossil fuel combustion, industrial waste, and various chemicals, urban soils are often enriched with trace metals (Wei and Yang, 2010; Luo et al., 2012). In contemporary urban soils, trace metal abundance is affected by an interaction between an anthropogenic effect, such as proximity to roadways, and local lithology (Fatoki, 1996; Yesilonis et al., 2008b; Argyraki and Kelepertzis, 2014). Vegetation modifies soil physico-chemical properties, differentially affecting trace metal retention; generally, trace metals accumulate through time in urban parks (Setälä et al., 2017). Trace metals such as Cu, Zn, Ni, and Fe are important cofactors for enzymes involved in a wide variety of metabolic processes such as methanogenesis (Mulrooney and Hausinger, 2003; Glass and Orphan, 2012), ammonia oxidation (Ensign et al., 1993; Ferguson, 1998), and denitrification (Woolfenden et al., 2013). In the soil environment, both pH and redox potential are driving factors that control the solubility of metals (Yesilonis et al., 2008a). At low availability, metals may be a limiting nutrient, yet at high availability they become toxic. We expect that urban microbes should have a higher abundance of heavy metal resistance genes to compensate for metal toxicity.

Evaluating the functional attributes of microbial communities is challenging. Many microbial ecology studies use highly conserved loci (such as 16S rRNA of the small subunit of prokaryotic ribosomes) to characterize the microbial community (Caporaso et al., 2012). These genes have been used to predict functional attributes of identified taxa (Langille et al., 2013). As with all phenotypic traits, it is in principle possible for taxonomically divergent organisms to converge in their phenotypes; and there is generally poor phylogenetic coherence of functional traits in bacteria (Philippot et al., 2010). Thus the small number of representative genomes used in PICRUSt may hamper the ability to accurately predict environmental samples in highly diverse soil communities. We addressed these deficits by annotating environmental DNA directly, using the program Metagenomic Rapid Annotation using the Subsystems Technology (MG-RAST) server (Glass et al., 2010). MG-RAST bins genes into functional groups. These groups may then be interrogated for convergence. Shotgun metagenomes of urban soils across five urban centers and four land-use types were used to determine if microbial community functional gene profiles converge. We chose to sequence and analyze DNA rather than RNA because we wanted to test the potential functionality of the soil community, rather than the activity of the community at a particular moment in time. We hypothesized that (1) genes related to N cycling (in both the oxidative and reductive pathways) would be more abundant in urban turf sites relative to the reference sites; (2) heavy metal resistance genes would be more abundant under turf and ruderal land-use; and (3) urban soil microbial community functional gene profiles would converge in urban turf and ruderal land-uses relative to reference sites.

## Materials and Methods

### Study Design

Soil sampling and DNA isolation are described in Epp Schmidt et al. (2017). Briefly, we collected soil cores (2.5 cm diameter and to a depth of 10 cm) in five cities. These cities range in climate from boreal-hemiboreal (Helsinki and Lahti, Finland) to humid subtropical (Baltimore, MD, United States), to continental (Budapest, Hungary) and to semiarid (Potchefstroom, South Africa) biomes. Soils were collected in the winter months of each city (November for Baltimore, Budapest, Helsinki and Lahti; July for Potchefstroom). Within each city, we sampled four land-use categories that were defined (Pouyat et al., 2017) as follows:

1. Reference: habitat that was beyond the city matrix, and that had minimal human impact; typically set aside for conservation and wildlife management purposes.
2. Remnant: habitat inside the urban matrix that was structurally similar to reference habitat, but not landscaped.
3. Turf: turfgrass/lawn system managed for aesthetic or functional purposes.
4. Ruderal: land that experienced a high degree of physical disturbance; typically construction or demolition sites.

For each land-use category we selected five replicate locations within each city; thus there were 20 samples per city (e.g., five locations per land-use category), and a total of 100 sampling locations. At each sampling location (representing one replicate of that land-use category), five random cores were taken from within a 3 m^2^ area. Any O_i_ (L) and O_e_ (F) horizons (organic horizons for which the origin of the organic matter can be distinguished; highly decomposed organic matter was retained) were removed, and the cores were composited and homogenized. Two grams of the homogenized soil material was added to 10 ml of LifeGuard RNA preservation solution (MoBio, Carlsbad, CA, United States).

### Sequence Library Preparation

The MOBIO Laboratories Inc., Powerlyzer^TM^ Powersoils^®^ DNA isolation kit (MoBio, Carlsbad, CA, United States) was used for the extraction and isolation of DNA. This was done according to the manufacture’s protocol (two minor exceptions are noted in Epp Schmidt et al., 2017). DNA was quantified using a QuBit^®^ 2.0 Fluorometer (Invitrogen, Carlsbad, CA, United States) and then diluted to 0.2 ng μL^–1^ for shotgun sequencing. The DNA library was prepared using the Nextera XT DNA Library Preparation Kit (Illumina, San Diego, CA, United States), following all kit protocols. Samples were indexed using the Nextera XT V2 96-sample indexing kit (Illumina, San Diego, CA, United States), and pooled for sequencing on an Illumina HiSeq 3000 (Oregon State University, Center for Genomics and Biocomputing, Corvalis, OR, United States). Not all samples would fit on a single run and some had low sequence depth (fewer than three million reads) after the first run, therefore, a second pooled library was sequenced at the same facility.

The gene quantities of ammonia monooxygenase subunit A (*amoA*) for ammonia oxidizing bacteria (AOB) and archaea (AOA) were determined by QPCR. Ammonia oxidizing bacteria were enumerated using forward primer 5′- GGGGTTTCTACTGGTGGT and reverse primer 5′-CCCCTCKGSAAAGCCTTCTTC (Rotthauwe et al., 1997). The AOAs were enumerated using forward primer 5′-GCARGTMGGWAARTTCTAYAA and reverse primer 5′-AAGCGGCCATCCATCTGTA (Mincer et al., 2007). We ran the QPCR on an Applied Biosystems Step One Plus Real-Time PCR System. The run method for AOA was 94°C for 15 min, then 45 cycles of: 94°C for 15 s; 52°C for 45 s; 72°C for 30 s; 78°C and acquisition for 15 s. The run method for AOB was 95°C for 1 min, then 40 cycles of: 95°C for 15 s; 58°C for 30 s; 72°C and acquisition for 60 s. Both methods were followed by a melt curve run of 95°C for 15 s; followed by a 0.3°C stepwise temperature ramp from 60°C to 95°C. For AOA, our amplification efficiencies for all plates fell between 96 and 105%. For AOB, amplification efficiencies were between 87 and 96%. We applied an efficiency correction as described in Hargreaves et al. (2013). We calculated that soils were amplifying at between 91 and 105% efficiency. We developed our standards from *Nitrosomonas europaea* (AOB) and an environmental clone (AOA) (Bowen, 2016).

### Trace Metal Quantification

Soil not added to the Lifeguard preservation solution was used in additional analyses. “Total” and “mobile” element concentrations were measured for four toxic heavy metals [nickel (Ni), zinc (Zn), cadmium (Cd), and cobalt (Co)]. Soil samples for analysis of inductively coupled plasma atomic emission spectroscopy (ICP-AES) were dried, ground and homogenized. For the analysis of nitrohydrochloric acid (aqua regia) soluble (“total”) microelement concentration (Baumann et al., 1992; MSZ 21470-50, 2006) 4.5 mL of concentrated hydrochloric acid (37% m/m), 1.5 mL of concentrated nitric acid (65% m/m) and 1 mL of hydrogen peroxide was added to 1 g of soil. Microwave digestion was carried out in a MLS-1200 Mega Lab station (Milestone Inc., Shelton, CT, United States). After digestion, the solution was cooled and filled up to 50 mL with double-distilled water. The “mobile” soil element fraction was measured in 0.5 M NH_4_-acetate + 0.02 M EDTA extract (Lakanen and Erviö, 1971). ICP-AES measurements were carried out on a ULTIMA 2 ICP optical emission spectrometer (HORIBA Jobin Yvon, Longjumeau, France) equipped with a Meinhart cyclonic spray chamber nebulizer with a demountable quartz torch; operating with high purity (99.999%) argon gas, at plasma and nebulizer gas flow rates of 12.0 and 0.70 L min^–1^, respectively, under 300 kPa nebulizer pressure, sample uptake rate of 1.0 mL min^–1^ with 30 s sample uptake delay. The temperature of the plasma was about 10000 K. The analysis was helped by an autosampler, with automata sample switch. Injection of the samples was carried out by continuous pumping, at a volume of 1 mL min^–1^. Measurement time was approx. 20 s/element. Reference wavelength for the AES was Ar, and the different wavelengths for analysis were chosen from the lowest to the highest. With this high resolution sequential ICP-AES, five elements were measured at wavelengths (nm) given in parentheses: Cd (228.802), Co (228.616), Ni (231.604), and Zn (213.856). Nitrate (NO_3_^–^N) and ammonia (NH_4_^+^-N) extracted by KCl were determined according to Bremner and Keeney (1965). Additional soil characteristics are described in Pouyat et al. (2015).

### Bioinformatics and Community Statistics

Sequences were analyzed using the quality control and sequence annotation pipeline MG-RAST (Glass et al., 2010). Tagmentation resulted in fragments that were between 1.5–2 kb in size. Therefore, forward and reverse reads were annotated separately and merged *a posteriori*. The MG-Rast filter for *Arabidopsis thaliana* was used to remove plant DNA, and the MG-Rast dereplication function was used to remove artificial duplicates. For dynamic trimming, the lowest acceptable phred score was set to 15; and the maximum allowable number of base pairs below this threshold was set to 5. Our data were downloaded and aggregated using MG-RAST default match parameters, with the exception that the maximum *e*-value was set to 1.0^∗^e^–15^ rather than 1.0^∗^e^–5^. Sequencing annotations were imported and summarized in R using an in-house pipeline that is freely available^1^. Because a single gene may contribute to multiple pathways, there was double-counting in the annotation process. We assumed that because any sequence that mapped to a particular gene would be annotated in the same fashion, double-counting would occur at the same rate across the dataset, and thus results would accurately reflect changes in the metagenome. The forward and reverse datasets and replicate sequencing runs were combined using the merge_samples function from the phyloseq (McMurdie and Holmes, 2013) package in R (R Core Team, 2015) by summing sample totals. We generated two shotgun sequence annotation datasets; one of functional annotations, the other of taxonomic assignments. The taxonomic assignments were also compared to a previous 16S rRNA amplicon study of the same samples (Epp Schmidt et al., 2017). After annotation and aggregation, the same statistical approaches were used for both the shotgun sequence and amplicon sequence libraries.

We previously published 16S rRNA amplicon data using QIIME as the bioinformatic pipeline (Epp Schmidt et al., 2017); there has since been some significant improvements to sequence identity inference. Namely, the implementation of the R package dada2 (Callahan et al., 2016) allowed us to infer amplicon sequence variants (ASV), a biologically relevant variant rather than an arbitrarily clustered group of similar sequences. Also, the SILVA database (Quast et al., 2013) offered an updated framework for annotating microbial taxonomy. Thus, we updated the 16S rRNA sequence annotation using a dada2-based pipeline, and 16S rRNA taxonomy annotations with SILVA. For dada2, the following parameters were used: forward reads were truncated at 200 bp, and the reverse reads at 150 pb. The first 18 bp were trimmed from the forward and 19 bp from the reverse reads to remove primers. Zero unknown bp were allowed; the expected error rate was set at two for both reads; and the truncQ parameter was also set to two. For removing bimeras, we used the “consensus” method. Taxonomy was assigned against SILVA v. 132. Note, we did not assign species names as in the dada2 tutorial.

We normalized our dataset by scaling counts to an external reference. For the amplicon dataset, we used 16S QPCR to scale our subsampling routine relative to 50000 sequences. For shotgun amplicon sequencing we used the total extracted DNA to scale our annotation relative to 6000000 sequences. In this way the abundance of taxa or gene counts in principle should reflect ecological differences in abundance. These normalized datasets were used in all downstream statistical analyses. We compared this method to using relative abundance, and found that relative abundance provided weaker correlation to environmental parameters, and lower power to detect effects on genes (Supplementary Figures S5, S6 and Supplementary Tables S2, S3). For differential abundance testing, we used the limma-voom (Law et al., 2014; Ritchie et al., 2015) method to make linear models for each function or taxon; and used *post hoc* tests to contrast remnant, turf and ruderal sites against reference to determine significant indicators of each environment. We chose the limma-voom package in particular because it does not require us to transform our data before doing statistical analysis. PERMANOVA analysis via the vegan package (Oksanen et al., 2016) was used to determine overall similarity of gene profiles across sites. We tested the whole metagenomic profile, and a number of gene subsets (Aromatic Carbon Degradation, Nitrite Reduction, Nx Reductases (a subset of all genes associated with N reduction), Nitrogenase, Antibiotic Resistance, Nickel Transport, Cobalt Transport, Arsenic Resistance, Copper Regulation, Cadmium Resistance, Copper-Zinc-Cobalt Resistance and Homeostasis, and Zinc Resistance) for multivariate convergence. These subsets were curated manually by selecting all gene ontologies that were related to the gene function. These definitions can be found in Table 1.

**TABLE 1:** Definition of manually curated subgroups of functional ontologies.

**Category**	**Definition**
Aromatic carbon degradation	All genes categorized under “Aromatic Carbon Degradation” of level 1 subsystems ontology
Nitrite reduction	All genes that include “nitrite reductase” or “nitrous-oxide reductase” in the level 4 subsystems description, including maturation and assembly proteins
Nx reductase	Same as the nitrite reduction group, but it excludes all genes that are categorized as being maturation or scaffold proteins
Nitrogenase	All genes that pertain to nitrogenase structure and function at the level 4 ontology, including assembly and maturation proteins
Nickel transport	Genes that are specific to Nickel transport such as Energy-Coupled-Factor transporters (ECF), and also genes that code for proteins that transport Ni along with other metals such as Co
Cobalt transport	All genes that are annotated as having Co transport at the level 4 ontology, including genes that cross-list Zn, Ni, and/or Mg transport
Antibiotics	Any level 4 ontology that includes “antibiotic resistance,” “multidrug resistance,” “multidrug efflux”, or “multidrug transport”
Arsenic resistance	All genes that include “arsenic resistance” in the level 4 subsystems ontology description
Copper regulation	All genes that are annotated as “copper resistance,” “copper tolerance,” “copper homeostasis,” “copper-translocating,” or “copper sensitivity”
Cadmium resistance	All cadmium efflux and transport genes, and includes cobalt-zinc-cadmium resistance proteins
CZC	Only genes that cross-list all three Cd, Zn and Co metal transport
Zinc resistance	All genes that are annotated to be involved with Zn transport, or are annotated as “Zn homeostasis” or “Zn resistance”

*These groups were tested for multivariate convergence.*

In order to test for convergence, we calculated a study-wide Bray-Curtis distance matrix for each dataset. We applied a multivariate homolog of the Levene’s test of homogeneity (Anderson, 2006) of variances as described in Epp Schmidt et al. (2017) to these ordinations. Briefly, we used the betadisper function from the vegan package to map samples into a two-dimensional ordinal space; then we used an ANOVA to compare the mean Euclidean distance-to-centroid within each land-use group to capture within-group dispersion. This test captures the structural diversity of the subset genes. To test the variation in estimated representation of each subset of genes, we used summed the total gene counts in each subset dataset, and subjected them to a Levene’s test using the car package V 3.0-2 (Fox and Weisberg, 2011) in R. We also used PERMANOVA in vegan to do a permutation-based ANOVA to test for differences between land-use for each subgroup. Using this procedure, we can use the count data from each gene as covariates in the model and estimate the variation that is explained by each gene in the model. We used this procedure to determine which Ni ECF genes were contributing most to Ni gene patterns across sites. An annotated version of the complete workflow in R can be found at https://github.com/djeppschmidt/GLUSEEN_2/.

## Results

Sequencing generated an average of 10 million sequences per sample. A total of 1.2 billion sequences were submitted to MG-RAST for annotation. On average, 10% of the sequences per sample did not pass quality filters; most that did not pass were filtered as duplicate sequences, an artifact of the sequencing preparation procedure (Supplementary Table S1). The annotation generated 3.4 billion seed subsystem database hits, which are classified into 12363 distinct subsystems ontology level 4 functional categories across all samples; 1141 distinct level 3 functional categories; and 21 distinct level 1 functional categories. After filtering for well-represented level 4 functions, we were left with 8765 functional ontologies for comparing city-wise differences in abundance, and 8493 functions for testing land-use effects. Previously published bacterial and archaeal 16S rRNA sequences from these same soil samples had 12.5 million total sequences (Epp Schmidt et al., 2017). Dada2 processing resulted in 56172 unique ASV. The limma-voom pipeline removed any taxa not broadly distributed across sites and categories. In microbial community studies that may not share many ASVs across site categories, this is a very stringent filter. In our case, it reduced the dataset from 56172 to 364 taxa. This filter was only applied for differential abundance testing; all other tests used the whole community.

**FIGURE 1:**
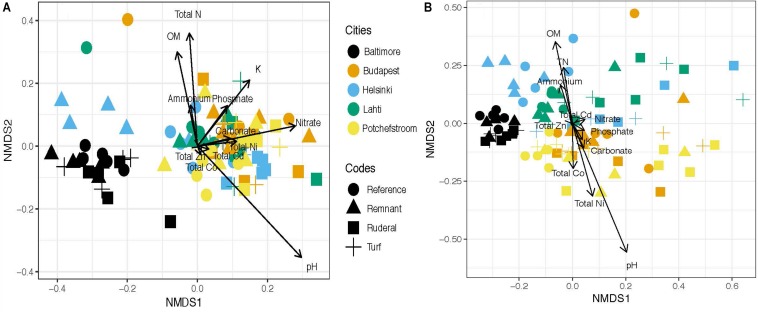
**(A)** NMDS ordination of the functional profile at MG-RAST level 4 subsystems ontology. Significant environmental covariates included: total N (*r*^2^ = 0.18, *p* = 0.002), pH (*r*^2^ = 0.16, *p* = 0.002), nitrate-N (*r*^2^ = 0.15, *p* = 0.003), potassium (*r*^2^ = 0.15, *p* = 0.001),% organic matter (OM) (*r*^2^ = 0.13, *p* = 0.006), and phosphate (*r*^2^ = 0.09, *p* = 0.03). **(B)** NMDS ordination of Bray–Curtis distance taxonomic assignments according to MG-RAST annotation. The significant covariates were pH (*r*^2^ = 0.61) and% OM (*r*^2^ = 0.42).

Cities were significantly different in respect to functional annotations (Figure 1A; PERMANOVA, *r*^2^ = 0.34, *P* = 0.001). Of the 8765 functional ontologies tested for differential abundance across cities, 8375 (or 95.6%) were significantly different (linear regression, *P* < 0.05). The taxonomic annotations also differed by land-use (Figure 1B; PERMANOVA, *r*^2^ = 0.37, *P* = 0.001). This is consistent with our previously published 16S rRNA amplicon data (Supplementary Figure S1), which also had significant differences between city (PERMANOVA *r*^2^ = 0.22, *P* = 0.001) and land-use (PERMANOVA *r*^2^ = 0.09, *P* = 0.001). It is also consistent with the differences in average soil parameters across cities (Table 2).

**TABLE 2:** The value of measured soil properties averaged by city with standard errors.

**Cities**	**pH**	**C_Org (g/100 g)**	**NH4_N (mg/kg)**	**NO3_N (mg/kg)**	**total_N (g/100 g)**
Baltimore	5.94 ± 0.23	2.41 ± 0.17	3.7 ± 0.266	1.88 ± 0.42	0.166 ± 0.0118
Budapest	7.14 ± 0.18	5.88 ± 1.12	9.92 ± 1.09	10.7 ± 2.02	0.488 ± 0.0783
Helsinki	5.36 ± 0.26	14 ± 2.85	27.6 ± 6.5	12.5 ± 2.72	0.677 ± 0.119
Lahti	5.45 ± 0.18	6.1 ± 1.14	13.9 ± 2.91	21.9 ± 4.66	0.358 ± 0.0504
Potchefstroom	6.51 ± 0.12	3.34 ± 0.43	14 ± 3.12	10.6 ± 4.41	0.277 ± 0.0321

*Nitrogen and carbon data are presented in either mg/kg or g per 100 g of soil.*

Neither metagenomic function or metagenomics-based taxonomy assignments converged due to land-use across cities (ANOVA, *F* = 0.0708, *P* = 0.97). Similarly, we found no convergence when N-cycling genes were examined in isolation (ANOVA: *F* = 0.169, *P* = 0.917). However, the subset of nitrogenase assembly, maturation and cofactor genes did converge according to land-use (ANOVA, *F* = 3.795, *P* = 0.0128), and a TukeyHSD test showed that turf is less variable than reference (adjusted *P* = 0.052) and remnant (adjusted *P* = 0.037) sites at a global scale. Also, the subset of nickel transport-related genes converged under urban land-use (ANOVA, *F* = 4.222, *P* = 0.008); there was significantly less variability under turf land-use compared to reference and remnant sites (ANOVA: within-group *N* = 20, *P* = 0.007; TukeyHSD, turf-reference *P* = 0.053; turf-remnant *P* = 0.029). One gene in particular, “ATPase component NikO of energizing module of nickel ECF transporter,” accounted for 12% of the total variation in the Ni transport subset. The following subsets of genes did not converge in structural similarity (ANOVA, *P* > 0.400), or abundance (Levene’s Test, *P* > 0.260) across urban land-use: Aromatic Carbon Degradation, Nitrite Reduction, Nx Reductases (a subset of all genes associated with N reduction), Antibiotic Resistance, Cobalt Transport, Arsenic Resistance, Copper Regulation, Cadmium Resistance, Copper-Zinc-Cobalt Resistance and Homeostasis, and Zinc Resistance. Land-use was a significant predictor of whole functional profiles (Figure 1A, PERMANOVA *r*^2^ = 0.05, *P* = 0.001), and interacted with city effects (Figure 1A, PERMANOVA *r*^2^ = 0.12, *P* = 0.052). Similarly, MG-RAST taxonomy assignments were significantly structured by both city (Figure 1B, PERMANOVA *r*^2^ = 0.37, *P* = 0.001) and land-use (Figure 1B, PERMANOVA *r*^2^ = 0.08, *P* = 0.001); and there was a significant interaction between city and land-use factors (Figure 1B, PERMANOVA *r*^2^ = 0.15, *P* = 0.001).

Of the 8491 annotated sequence ontologies that passed the filter, 160 were significantly higher in proportional abundance in turf sites relative to reference sites, while 13 were significantly lower in proportional abundance. Ruderal sites had 274 genes that were significantly higher in relative abundance compared to reference sites, and 31 genes that had significantly lower relative abundance compared to reference sites. The ammonia monooxygenase gene and several nitrite reductase-associated genes were significantly higher in turf sites (Table 3). Turf sites also had higher proportions of several Ni transport and Cu resistance genes compared to reference sites (Table 4). There was a higher relative abundance of both the reduction and oxidation of N species (Table 3), and several genes associated with the oxidation of H_2_ (Table 4) under turf and ruderal land-use. Two genes associated with antibiotic resistance proteins in the MarA transcriptional regulator site, and one gene associated with the MexD multidrug efflux pump were higher relative abundance in ruderal sites (Table 5). Other genes with significantly higher relative abundance in turf or ruderal included Ni and Co transport and resistance, Cu resistance, and Zn resistance genes (for a list of all significant indicators of land-use, see Supplementary Information). Remnant sites had 80 genes with increased relative abundances compared to reference sites, and two genes that were lower. None of the indicator genes in the remnant sites were directly involved in NH_3_ oxidation, NO_2_^–^ reduction, Cu resistance and transport, Zn resistance and transport, Co transport, Ca transport, or antibiotic resistance (Supplementary Information).

**TABLE 3:** Functional ontologies associated with nitrate, nitrite, nitrous oxide reductase or reductase maturation, and ammonia oxidation.

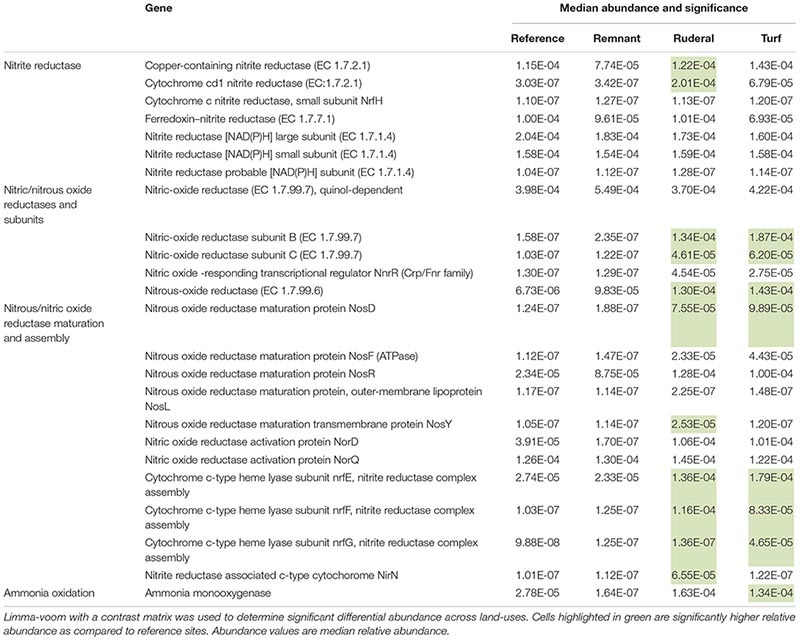

*Limma-voom with a contrast matrix was used to determine significant differential abundance across land-uses. Cells highlighted in green are significantly higher relative abundance as compared to reference sites. Abundance values are median relative abundance.*

**TABLE 4:** Functions associated with Ni, Co, Cd, Zn transport or resistance; Ni-Fe hydrogenases and support proteins.

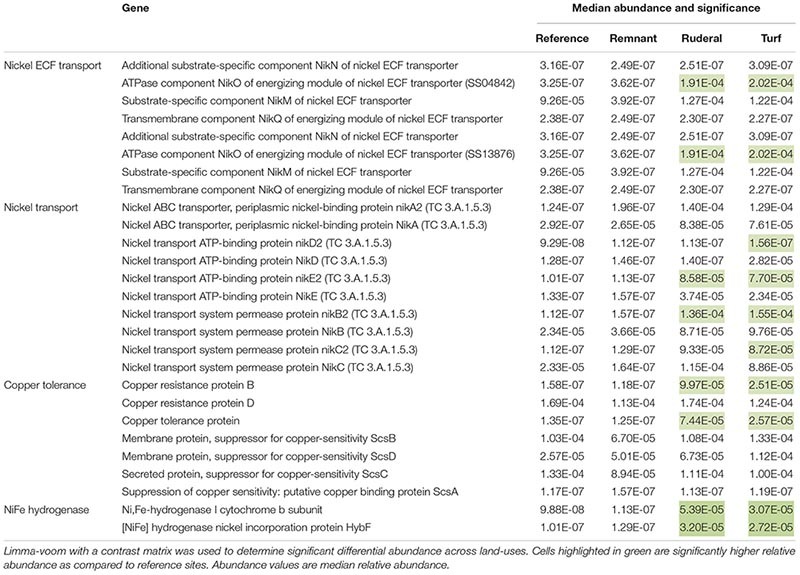

*Limma-voom with a contrast matrix was used to determine significant differential abundance across land-uses. Cells highlighted in green are significantly higher relative abundance as compared to reference sites. Abundance values are median relative abundance.*

**TABLE 5:** All functional genes associated antibiotic resistance.

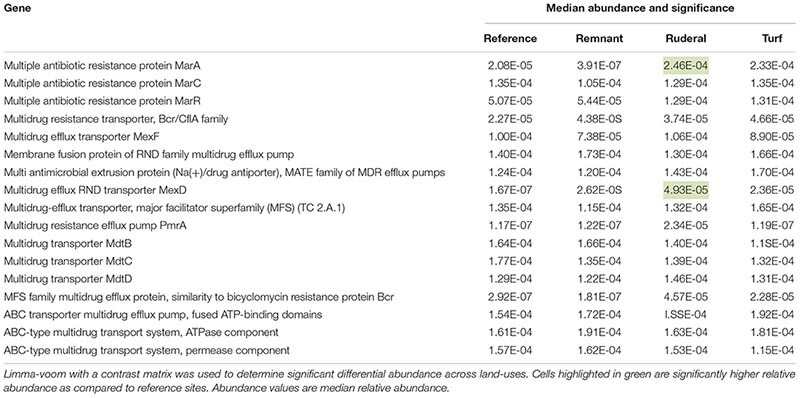

*Limma-voom with a contrast matrix was used to determine significant differential abundance across land-uses. Cells highlighted in green are significantly higher relative abundance as compared to reference sites. Abundance values are median relative abundance.*

**FIGURE 2:**
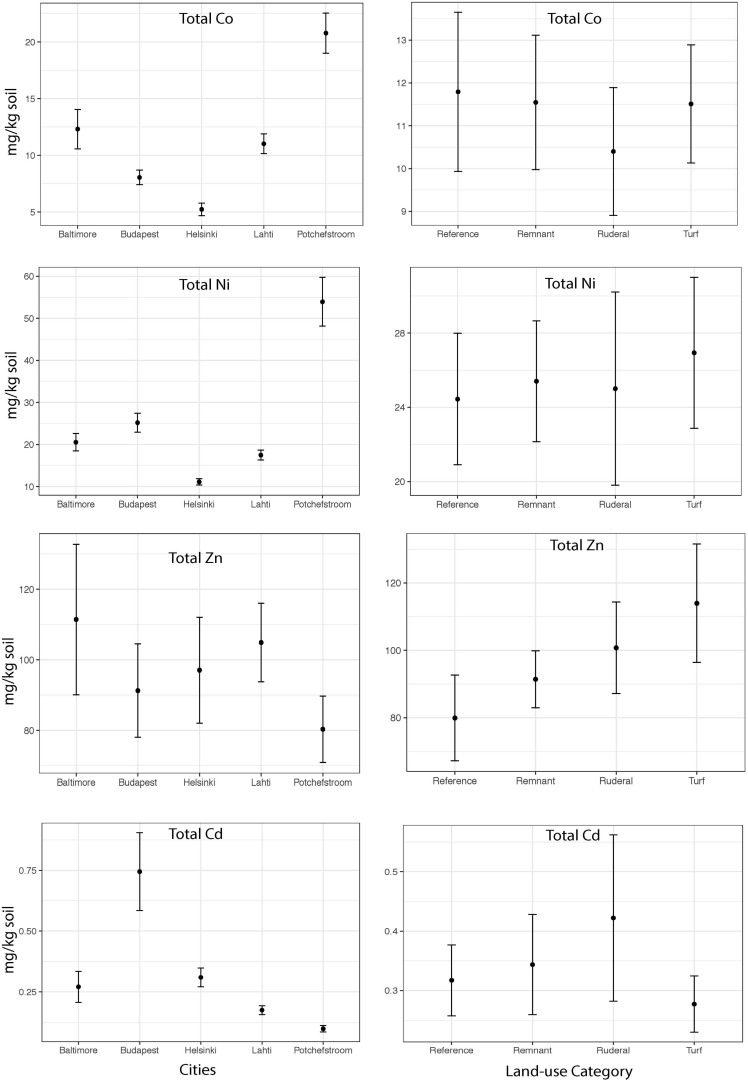
The mean concentrations of total Co, Cd, Ni and Zn (mg of metal kg soil^–1^) within each city and the mean concentration of total Co, Cd, Ni and Zn (mg of metal kg soil^–1^) for each land-use averaged across all cities.

**FIGURE 3:**
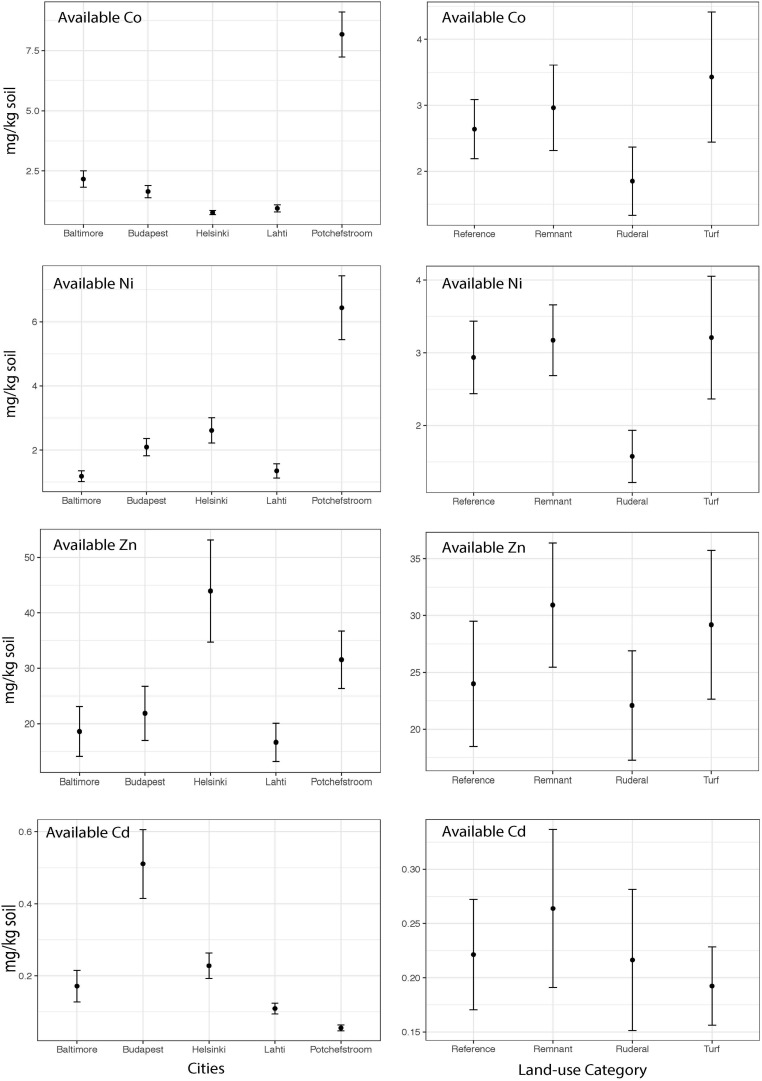
The mean concentrations of available Co, Cd, Ni and Zn (mg of metal kg soil^–1^) within each city and the mean concentration of available Co, Cd, Ni and Zn (mg of metal kg soil^–1^) for each land-use averaged across all cities.

The heavy metal extraction method estimates biologically available heavy metals using a weak acid extraction, and total heavy metals using a strong acid digestion. We will use the words “available” and “total” to refer to the respective extraction methods. There is a high degree of correlation between the two extraction methods for Cd (Pearson *r* = 0.92), but lower correlation for the other heavy metals (*r* = 0.62–0.70). Except for Zn, total heavy metal abundances were significantly different between cities (Figure 2; Cd: ANOVA *F* = 8.8, *P* = 0.001; Co *F* = 22.1, *P* = 0.001; Ni *F* = 32.3, *P* = 0.001; Zn *F* = 0.65, *P* = 0.6). Total Co and Ni were significantly more abundant in Potchefstroom (South Africa) than in any other city (Figure 2; Co two-way ANOVA city *P* = 0.001; Ni two-way ANOVA city *P* = 0.001), whereas Cd was significantly more abundant in Budapest than in the other cities (Figure 2; two-way ANOVA city *P* = 0.001). Total Zn did not differ either between cities, or across land-use types (Figure 2, two-way ANOVA city *P* = 0.6; land-use *P* = 0.3). Available Zn was significantly different across cities but not land-use (Figure 3, city *F* = 3.9, *P* = 0.006; land-use *F* = 0.69, *P* = 0.56). None of the heavy metals, either total or available, converged based on our urban land-use categories (Levene test; all *F* < 1.2, all *P* > 0.36). We found no effect of land-use on total Cd, Co, Ni, or Zn (Figure 2, ANOVA Cd *F* = 0.7, *P* = 0.5; Co *F* = 0.018, *P* = 0.99; Ni *F* = 0.70, *P* = 0.56; Zn *F* = 1.22, *P* = 0.31). In contrast, available Ni and Co were both significantly different across land-use (Ni *F* = 3.25, *P* = 0.027; Co *F* = 2.99, *P* = 0.037). Available Ni and Co were both lower in ruderal sites (Figure 3).

**FIGURE 4:**
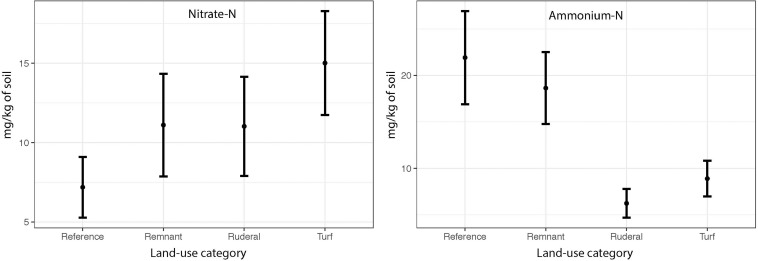
The mean NO_3_-N and NH_4_-N concentrations (mg-N kg soil^–1^) by land use across all cities.

The NO_3_-N did not differ between land-uses (Figure 4: ANOVA *F* = 1.04, *P* = 0.38) and did not converge (Levene’s Test *F* = 0.886, *P* = 0.45). However, we did find significant differences in NH_4_^+^-N between land-uses (Figure 4: ANOVA, *F* = 12.3, *P* = 0.001) and a significant convergence in turf (Levene’s test *F* = 4.29, *P* = 0.007). In all cities except Potchefstroom, mineral ammonia availability was highest in reference and remnant sites, and much lower in ruderal and turf sites (data not shown).

**FIGURE 5:**
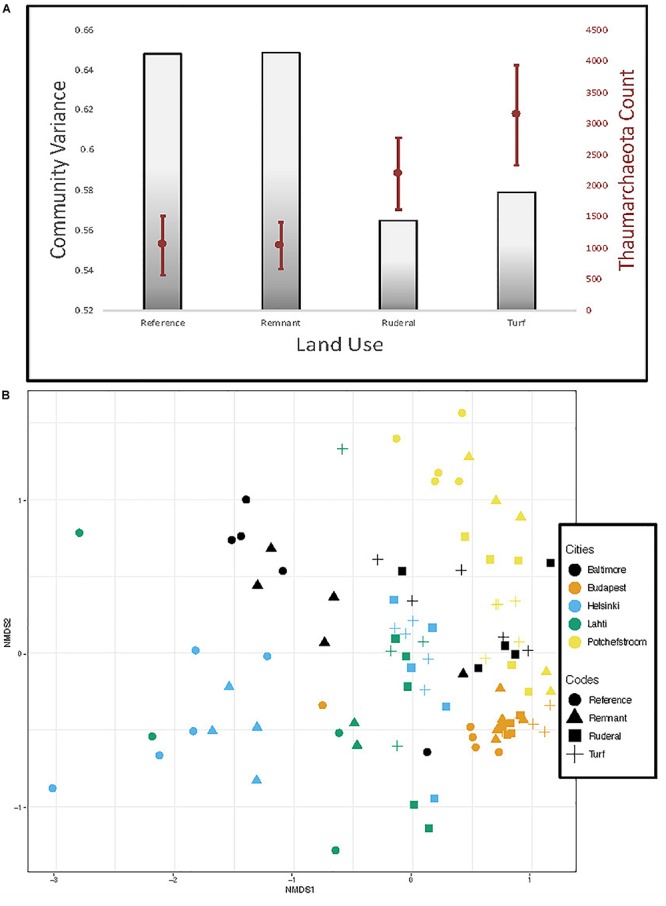
**(A)** Gray bars show variance in amplicon-based archaeal communities and overlaid in red is the abundance of sequences matching Thaumarchaeota. **(B)** NMDS of archaeal communities based on 16S rRNA sequencing and analysis with the dada2 pipeline.

**FIGURE 6:**
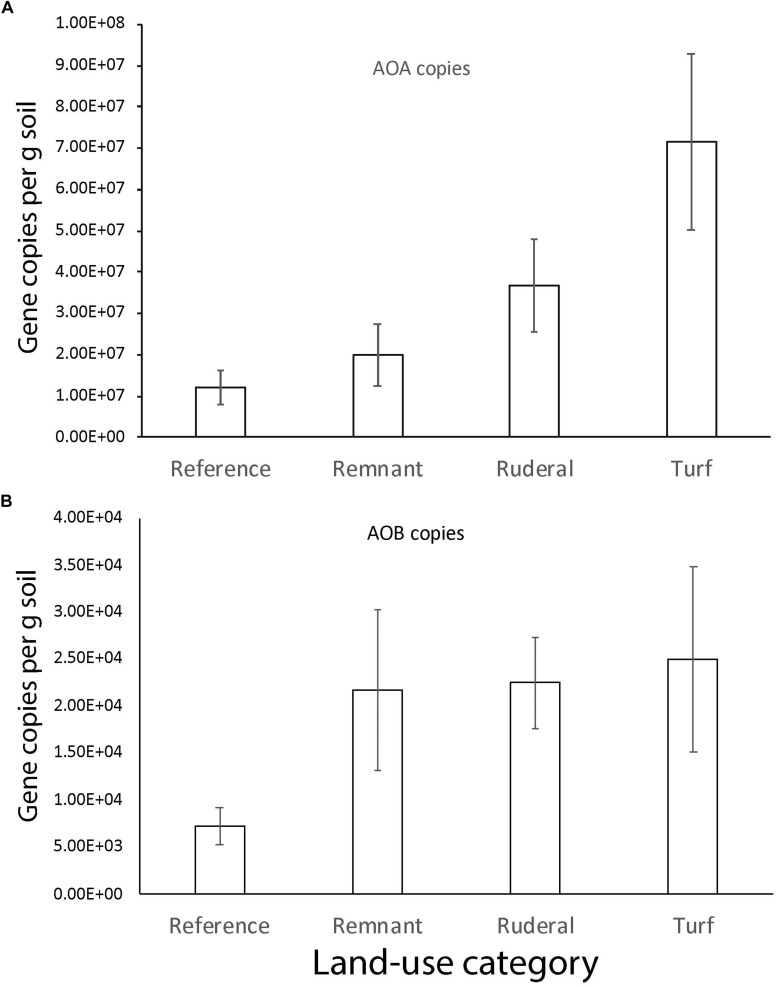
The number of gene copies (g of soil^–1^) for **(A)** archaeal and **(B)** bacterial *amoA*.

A reanalysis of the 16S rRNA data using dada2 ASV resulted in a stronger archaeal convergence pattern than we previously reported (Figure 5; Epp Schmidt et al., 2017). Archaea converged significantly in turf (Figure 5B; TukeyHSD, *P* = 0.004) and ruderal (Figure 5B; TukeyHSD, *P* = 0.003) sites with respect to reference sites. Convergence was largely driven by ammonia-oxidizing archaea in the phylum *Thaumarchaeota*, which were significantly higher in abundance under turf and ruderal land (Figure 5; adj. *P* = 0.001). The AOA *amoA* gene copy numbers were more abundant in turf and ruderal sites than in reference sites (Figure 6; *F* = 4.9, *P* = 0.002). In contrast, the AOB *amoA* gene copy numbers were not statistically different (*F* = 2.165, *P* = 0.08) though the pattern was similar. There was a relatively high correlation between log-transformed 16s rRNA sequence abundance of putatively identified ammonia-oxidizing archaea (i.e., members of the phylum *Thaumarchaeota*) and log-transformed *amoA* gene abundance (Supplementary Figure S2; Pearson’s *r* = 0.78, *P* < 0.001); and a weaker correlation in putatively identified ammonia-oxidizing bacteria (members of the genera *Nitrosomonas*, *Nitrococcus*, and *Nitrospira*) after a similar transformation (*r* = 0.37, *P* < 0.001). There was no relationship between NH_4_^+^ or NO_3_^–^ -N concentrations and the abundance of either AOA (data not shown, Pearson: NH_4_^+^ *r* = −0.08; NO_3_ *r* = −0.09) or AOB (data not shown, Pearson: NH_4_^+^ *r* = −0.19; NO_3_ *r* = 0.10); but a weak correlation between the log-transformed ammonia-oxidizing gene copy numbers and log-transformed relative abundance of ammonia monooxygenase sequence annotations (Supplementary Figure S3; Pearson *r* = 0.63). There was a bi-modal relationship where very low representation of ammonia oxidation genes in the shotgun dataset did not correspond well to the abundance of *amoA* genes quantitated using QPCR. It was not possible to run limma-voom on the archaeal community because there were too many sites with little or no archaea. In order to test significance of archaeal indicators, we ran the archaeal community with the bacterial community, and noted when indicator species were archaeal. While no single methanogenic taxon was significantly associated with either turf or ruderal sites, the aggregated abundance of putatively identified archaeal methanogens in our study (*Methanobacteria*, and *Methanomicrobia*) was significantly higher in ruderal sites compared to remnant (ANOVA: *F* = 2.77, *P* = 0.046; TukeyHSD: ruderal-remnant *P* = 0.054), and to a lesser extent reference (TukeyHSD: ruderal-reference *P* = 0.089; Supplementary Figure S4).

## Discussion

We found only two groups of genes that converged when they were isolated from the rest of the gene profiles: nickel transport and nitrogenase genes. Unlike taxonomy data, the gene profiles did not converge when more groups of genes were considered together. This could be due to a: (1) functional redundancy of housekeeping genes across cities, such as those involved in DNA replication or protein synthesis, that are ubiquitous across a wide variety of microorganisms (Myrold et al., 2014); (2) substantial differences of gene profiles between cities, suggesting that soil properties are a strong selecting factor, and (3) current metagenomic databases are limited in their ability to assign function to soil microorganisms. Nonetheless, we found strong evidence that convergence of the taxonomy was consistently associated with similar changes in metabolic strategy across cities. In sites where we found higher abundances of putatively identified ammonia oxidizers, we also found higher abundance of ammonia oxidizing genes in both our QPCR and shotgun metagenome datasets. In sites that we found higher abundance of putatively identified methanogens, we also found higher relative abundance of methanogenesis-related genes, and a convergence of nickel transport genes that was likely related to methanogenesis.

### Nitrogen Metabolism

The archaeal community composition did converge according to 16S rRNA amplicon sequencing (Figure 5B; Epp Schmidt et al., 2017). In particular, this convergence was driven by an enrichment in ammonia-oxidizing archaea (Figure 5B). There was a higher relative abundance of sequences matching ammonia-oxidizing archaea (*Thaumarchaeota*) (Figure 5B; Brochier-Armanet et al., 2008) and nitrite-oxidizing bacteria (*Nitrospira*) (data not shown, Daims et al., 2015) in turf, and higher *amoA* gene copy numbers (Figure 6). These results point to the enrichment of the nitrifying community particularly in turf sites and agree with studies that have measured increased nitrification rates in urban areas (Reisinger et al., 2016). Although there was not convergence *per se* (i.e., less variability in *amoA* gene quantity in all turf sites, compared to more variability in other land-use categories), there was an increase in *amoA* genes and nitrifier sequence abundance in all turf sites compared to their corresponding reference sites within each city. In addition to nitrification, other N oxidative and reductive reactions were enriched in the urbanized land-use. Genes associated with dissimilatory N reduction and denitrification were more abundant in turf and ruderal sites, including: N_2_O reductase, NO reductase, NO_2_ reductase genes, and NO_x_ reductase maturation genes (Table 3). In particular, the nitrous oxide reductase (*Nos* genes) and nitric oxide reductase (*Nor* genes) were enriched, as were genes that are associated with cytochrome C and Ferredoxin (*nrf* genes). These results suggest that the more disturbed and managed ruderal and turf sites contain more microbes capable of dissimilatory nitrate reduction and denitrification, but quantitative approaches such as QPCR and potential assays are needed to confirm this observation.

There is broad consensus in the literature that urban areas experience N enrichment through a number of mechanisms, including the atmospheric deposition of N (Galloway et al., 1994). Our data suggests that the biological production and consumption of NH_4_^+^ are each likely enriched under urban turf sites. A key question is whether these biological effects will be balanced. Turf sites have on average lower organic carbon than reference sites (and therefore lose cation exchange sites), which will contribute to a lower capacity of the soils to hold NH_4_^+^. This would limit the stable pool of NH_4_^+^ adsorbed to the surface of soil colloids. We think it is likely that the reduction of NH_4_^+^ under turf sites is a result of both a physical and biological processes. In our dataset, soil NH_4_^+^-N and NO_3_^–^-N pools were not correlated to the abundance of N-cycling genes. However, our mineral N data were one-time measurements of N pools and not reflective of the total yearly N flux. To determine whether the biological or physical edaphic factors are controlling the NH_4_^+^-N pool, more detailed data on gross and net N flux rates are needed. If it is confirmed that the pools are at least partially controlled by microbial process, it would represent a mechanism by which microbial community dynamics contribute to the convergence of the urban ecosystem.

### Trace Metals and Methanogenesis

There was evidence of both increased trace metal resistance and trace metal use in urban soils. For example, we found higher abundance of Ni resistance and Ni transport genes, along with resistance genes for several trace metals, including Cu and Zn under ruderal land-use (Supplementary Information). While many trace metals are primarily derived from the parent material, vehicles are associated with creating hotspots of Cu, Zn, and Ni enrichment in the environment (Yesilonis et al., 2008b). The higher abundance of these resistance genes would appear to support the hypothesis that there may be some effect of land-use on exposure to these trace metals. However, our data on Zn and Ni availability do not provide compelling support for high toxicity of either trace metal in ruderal sites; we found lower Ni availability under ruderal sites where we also found higher relative abundance of Ni resistance genes. We do not have data on Cu availability or abundance.

Our data suggests that the convergence of Ni transport genes in turf sites, and the increased prevalence of Ni transport genes in turf and ruderal sites is in part related to an increase in prevalence of methanogens. Nickel is a particularly important cofactor for methanogenesis (Mulrooney and Hausinger, 2003; Glass and Orphan, 2012), and energy coupled factor (ECF) transport genes are associated with nutrient capture (Kirsch and Eitinger, 2014). We found a higher relative abundance of Ni ECF transport genes in turf and ruderal sites, along with two genes that are involved in catalyzing methanogenesis (i.e., NiFe hydrogenase and NiFe hydrogenase maturation) in turf sites (Table 4). The abundance of putative methanogens was also significantly higher in ruderal sites compared to other land-uses (Supplementary Figure S4). There is remarkable concordance between the pattern of abundance for Ni-Fe hydrogenase, Ni ECF transport, convergence of Ni transport genes, and putative methanogens under turf and ruderal land-use. This suggests that a greater abundance of methanogens at least partially accounts for the higher relative abundance of Ni transport genes under both ruderal and turf land-use. Disturbance in ruderal sites creates a higher prevalence of anaerobic microsites by destroying the soil structure; perhaps creating anaerobic microsites that would be ideal habitat for methanogens. A natural inference given this data is that the urban soil microbial community is likely producing more methane. Urban soils have been reported to have lower rates of methane uptake (Kaye et al., 2004; Groffman and Pouyat, 2009), but gross methanogenesis has not been measured. If rates of gross methanogenesis were higher in urban soils, this could have accounted for lower net consumption of methane observed within these laboratory incubations.

### Antibiotic Resistance

The multiple antibiotic resistance gene marA (Table 5) is a transcriptional regulator isolated from *E. coli*. It regulates a number of chromosomal genes that include antibiotic resistance, virulence, but also acid tolerance (Ruiz et al., 2008). It is not clear what specific role it is playing in the context of the urban soil environment. The marC and marR genes were also identified in our samples but were not significantly different across land-use types. mexD multidrug efflux pumps are particularly well studied in the context of *Pseudomonas aeruginosa* pathogenicity, and have been shown to be upregulated in the presence of low pH, high bacterial concentrations, or stationary growth phase (Aeschlimann, 2003). However, we can find no papers on its role and behavior in soil systems.

### Qualifications

Within the metagenomic analysis and interpretation presented here, we have taken care not to conflate relative gene abundance in shotgun sequence data as actual abundance in the environment unless we have independent data that corroborate this interpretation. Amplicon sequencing methodologies are able to achieve near quantitative performance of the entire sequencing methodology by applying a *post hoc* abundance correction using an independent measure of total abundance (preprint: Jian et al., 2018). We used a similar method using scaled subsampling in order to achieve a more accurate gene abundance estimate that is robust against artifacts of sequencing depth (Supplementary Figures S5, S6 and Supplementary Tables S1, S2). However, there were still apparent artifact in our qualitative (sequenced) counts, suggesting there may still be annotation bias. The development of a standard protocol and the proper analytical approaches for mitigating these artifacts should be considered a key objective for future research to promote high quality environmental microbiome research.

## Conclusion

Functional gene profiles of the microbiome did not converge under urban land-use; but there was evidence of functional differences within subpopulations of the microbial community. For example, archaeal convergence was primarily driven by the proliferation of ammonia-oxidizers under turf and ruderal land-use. Increased populations of ammonia-oxidizers may contribute to the convergence of soil NH_4_^+^ pools within turf and ruderal land-use. Some trace metal resistance genes are enriched under turf and ruderal land-use; but we also found evidence that higher Ni transport gene abundances could be driven by an enrichment of methanogens. Our results also demonstrate that functional redundancy may obscure ecologically relevant patterns that are occurring within subgroups of the data. To our knowledge this is the first reported shotgun metagenomic analysis of urban soils. These data demonstrate the usefulness of soil metagenomics in providing data on numerous processes simultaneously, but also the limitations of these methods and the necessity to use these data as a first step toward hypothesis building and more comprehensive analysis of these urban systems.

## Author Contributions

DE conducted the DNA extraction, quantification, sequence library preparation, annotation, data analysis, and manuscript preparation. KS is the PI of the grant, designed the study, and selected the sites in Baltimore. RP, HS, DK, EH, SC, IY, and ZT designed the study, selected the sites, and participated in soil sampling. MD participated in soil sampling and provided heavy metal data on soils. SY designed the study, oversaw all of the lab work, and bioinformatics and data analysis. All authors discussed the results and commented on the manuscript.

## Conflict of Interest

The authors declare that the research was conducted in the absence of any commercial or financial relationships that could be construed as a potential conflict of interest.

## Footnotes

1 https://github.com/Djeppschmidt/mgrastr
