# Supplementary material for: Metagenomics Reveals Bacterial and Archaeal Adaptation to Urban Land-Use: N Catabolism, Methanogenesis, and Nutrient Acquisition
Source: Front Microbiol. 2019 Oct 10;10:2330. doi: 10.3389/fmicb.2019.02330 (PMC6795690; doi:10.3389/fmicb.2019.02330)
Supplement: Supplementary file 1 [file Data_Sheet_1.PDF]

Supplemental Information:

Lists of functions and taxa that have a significant response to land use.

Functions:

Higher abundance under remnant:

[1] "Serine hydroxymethyltransferase (EC 2.1.2.1)66"
[2] "Acetoin dehydrogenase E1 component beta-subunit (EC 1.2.4.-)124"
[3] "Peptide chain release factor 1253"
[4] "Peptide chain release factor 1719"
[5] "Aspartyl-tRNA synthetase (EC 6.1.1.12)793"
[6] "Signal recognition particle, subunit Ffh SRP54 (TC 3.A.5.1.1)1050"
[7] "NAD-dependent glyceraldehyde-3-phosphate dehydrogenase (EC 1.2.1.12)1388"
[8] "Holo-[acyl-carrier protein] synthase (EC 2.7.8.7)1647"
[9] "Adenylosuccinate synthetase (EC 6.3.4.4)1840"
[10] "Seryl-tRNA synthetase (EC 6.1.1.11)2055"
[11] "1-hydroxy-2-methyl-2-(E)-butenyl 4-diphosphate synthase (EC 1.17.7.1)2187"
[12] "Propionyl-CoA carboxylase beta chain (EC 6.4.1.3)2206"
[13] "Peptide methionine sulfoxide reductase MsrA (EC 1.8.4.11)2548"
[14] "Coenzyme PQQ synthesis protein E2771"
[15] "1-hydroxy-2-methyl-2-(E)-butenyl 4-diphosphate synthase (EC 1.17.7.1)2885"
[16] "Lipoate synthase3101"
[17] "Sulfate adenylyltransferase subunit 2 (EC 2.7.7.4)3396"
[18] "Acetoin dehydrogenase E1 component beta-subunit (EC 1.2.4.-)3452"
[19] "Adenylosuccinate lyase (EC 4.3.2.2)3490"
[20] "6-phosphogluconate dehydrogenase, decarboxylating (EC 1.1.1.44)3615"
[21] "NAD-dependent glyceraldehyde-3-phosphate dehydrogenase (EC 1.2.1.12)4140"
[22] "Phosphogluconate dehydratase (EC 4.2.1.12)4145"
[23] "Serine hydroxymethyltransferase (EC 2.1.2.1)4285"
[24] "Acetyl-coenzyme A carboxyl transferase alpha chain (EC 6.4.1.2)4382"
[25] "Holo-[acyl-carrier protein] synthase (EC 2.7.8.7)4393"
[26] "Serine hydroxymethyltransferase (EC 2.1.2.1)5046"
[27] "Seryl-tRNA synthetase (EC 6.1.1.11)5048"
[28] "Serine hydroxymethyltransferase (EC 2.1.2.1)5053"
[29] "Thioredoxin reductase (EC 1.8.1.9)5073"
[30] "NAD-dependent glyceraldehyde-3-phosphate dehydrogenase (EC 1.2.1.12)5125"
[31] "Isocitrate lyase (EC 4.1.3.1)5169"
[32] "Sulfate adenylyltransferase subunit 2 (EC 2.7.7.4)5550"
[33] "1-deoxy-D-xylulose 5-phosphate synthase (EC 2.2.1.7)5750"
[34] "1-hydroxy-2-methyl-2-(E)-butenyl 4-diphosphate synthase (EC 1.17.7.1)5751"
[35] "Lipoate synthase6046"
[36] "Peptide chain release factor 16205"
[37] "Serine hydroxymethyltransferase (EC 2.1.2.1)6208"

[38] "tRNA uridine 5-carboxymethylaminomethyl modification enzyme GidA6799"
[39] "Periplasmic aromatic aldehyde oxidoreductase, molybdenum binding subunit YagR6826"
[40] "Glutamine amidotransferase chain of NAD synthetase6962"
[41] "6-phosphogluconate dehydrogenase, decarboxylating (EC 1.1.1.44)7457"
[42] "Peptide methionine sulfoxide reductase MsrA (EC 1.8.4.11)7471"
[43] "Serine hydroxymethyltransferase (EC 2.1.2.1)7976"
[44] "Serine hydroxymethyltransferase (EC 2.1.2.1)8359"
[45] "Adenylosuccinate lyase (EC 4.3.2.2)8601"
[46] "Adenylosuccinate synthetase (EC 6.3.4.4)8602"
[47] "Inosine-5'-monophosphate dehydrogenase (EC 1.1.1.205)8615"
[48] "Peptide methionine sulfoxide reductase MsrA (EC 1.8.4.11)8679"
[49] "1-deoxy-D-xylulose 5-phosphate synthase (EC 2.2.1.7)8719"
[50] "NAD-dependent glyceraldehyde-3-phosphate dehydrogenase (EC 1.2.1.12)8724"
[51] "Thioredoxin reductase (EC 1.8.1.9)8751"
[52] "Coenzyme PQQ synthesis protein E8785"
[53] "NAD-dependent glyceraldehyde-3-phosphate dehydrogenase (EC 1.2.1.12)8972"
[54] "NADH-ubiquinone oxidoreductase chain F (EC 1.6.5.3)9040"
[55] "tRNA uridine 5-carboxymethylaminomethyl modification enzyme GidA9462"
[56] "Serine hydroxymethyltransferase (EC 2.1.2.1)9717"
[57] "Isocitrate lyase (EC 4.1.3.1)9743"
[58] "Propionyl-CoA carboxylase beta chain (EC 6.4.1.3)9758"
[59] "Serine hydroxymethyltransferase (EC 2.1.2.1)9763"
[60] "Succinyl-CoA ligase [ADP-forming] alpha chain (EC 6.2.1.5)9770"
[61] "Phosphoglucosamine mutase (EC 5.4.2.10)9823"
[62] "6-phosphogluconate dehydrogenase, decarboxylating (EC 1.1.1.44)10391"
[63] "Succinyl-CoA ligase [ADP-forming] alpha chain (EC 6.2.1.5)10607"
[64] "Thioredoxin reductase (EC 1.8.1.9)10797"
[65] "Peptide chain release factor 111041"
[66] "Aspartyl-tRNA synthetase (EC 6.1.1.12)11199"
[67] "Seryl-tRNA synthetase (EC 6.1.1.11)11255"
[68] "Tyrosyl-tRNA synthetase (EC 6.1.1.1)11265"
[69] "tRNA uridine 5-carboxymethylaminomethyl modification enzyme GidA11411"
[70] "tRNA uridine 5-carboxymethylaminomethyl modification enzyme GidA11428"
[71] "tRNA uridine 5-carboxymethylaminomethyl modification enzyme GidA11469"
[72] "Signal recognition particle, subunit Ffh SRP54 (TC 3.A.5.1.1)11540"
[73] "Phosphoglucosamine mutase (EC 5.4.2.10)11774"
[74] "Signal recognition particle, subunit Ffh SRP54 (TC 3.A.5.1.1)11803"
[75] "Thioredoxin reductase (EC 1.8.1.9)12081"
[76] "Coenzyme PQQ synthesis protein E12194"
[77] "Lipoate synthase12231"
[78] "NADH-ubiquinone oxidoreductase chain F (EC 1.6.5.3)12233"
[79] "Peptide chain release factor 112301"
[80] "Serine hydroxymethyltransferase (EC 2.1.2.1)12306"

Lower abundance under remnant:

[1] "Predicted transcriptional regulator of the myo-inositol catabolic operon5579"
[2] "Triacylglycerol lipase precursor (EC 3.1.1.3)11165"

Higher abundance under turf:

[1] "2,3-butanediol dehydrogenase, S-alcohol forming, (S)-acetoin-specific (EC 1.1.1.76)121"
[2] "Thiosulfate reductase electron transport protein phsB485"
[3] "Thiosulfate reductase precursor (EC 1.-.-.-)486"
[4] "Arsenical pump-driving ATPase (EC 3.6.3.16)652"
[5] "Carnitine operon protein CaiE1548"
[6] "Electron transfer flavoprotein, beta subunit FixA1555"
[7] "Transcriptional activatory protein CaiF1557"
[8] "Putative glycosyltransferase protein1823"
[9] "Chitodextrinase precursor (EC 3.2.1.14)2310"
[10] "Sodium-Choline Symporter2413"
[11] "Chorismate synthase (EC 4.2.3.5)2453"
[12] "2,3-dihydroxy-2,3-dihydro-phenylpropionate dehydrogenase (EC 1.3.1.-)2467"
[13] "3-phenylpropionate dioxygenase, beta subunit (EC 1.14.12.19)2473"
[14] "Mhp operon transcriptional activator2480"
[15] "Cobalamin synthase2606"
[16] "Cation efflux system protein CusC precursor2622"
[17] "Copper sensory histidine kinase CusS2630"
[18] "Additional substrate-specific component CbiN of cobalt ECF transporter2677"
[19] "ATPase component CbiO of energizing module of cobalt ECF transporter2684"
[20] "Cobalamin synthase2692"
[21] "ABC transporter, ATP-binding protein YnjD2778"
[22] "ABC transporter, permease protein YnjC2780"
[23] "DedA family inner membrane protein YdjX2782"
[24] "Putative phosphatidylglycerophosphate synthase2790"
[25] "FIG002577: Putative lipoprotein precursor2967"
[26] "Colanic acid biosysnthesis glycosyl transferase WcaI3041"
[27] "Chorismate synthase (EC 4.2.3.5)3079"
[28] "Inner membrane protein YhaI3222"
[29] "Copper sensory histidine kinase CusS3292"
[30] "Minor curlin subunit CsgB, nucleation component of curlin monomers3359"
[31] "D-allose ABC transporter, substrate-binding component3433"
[32] "Transcriptional regulator of D-allose utilization, RpiR family3441"
[33] "DedA family inner membrane protein YdjX3443"
[34] "DedA family inner membrane protein YdjZ3444"
[35] "Nitric-oxide reductase subunit B (EC 1.7.99.7)3476"
[36] "Nitric-oxide reductase subunit C (EC 1.7.99.7)3477"
[37] "Nitrous-oxide reductase (EC 1.7.99.6)3481"
[38] "Nitrous oxide reductase maturation protein NosD3483"
[39] "Predicted nucleoside ABC transporter, ATP-binding component3530"
[40] "Predicted nucleoside ABC transporter, permease 1 component3531"
[41] "Pectinesterase (EC 3.1.1.11)3590"

[42] "Positive regulator of L-idonate catabolism3635"
[43] "LysR family transcriptional regulator YfiE3713"
[44] "Predicted nucleoside ABC transporter, ATP-binding component3970"
[45] "Predicted nucleoside ABC transporter, permease 1 component3971"
[46] "PTS system, sorbose-specific IIB component (EC 2.7.1.69)3995"
[47] "PTS system, sorbose-specific IIC component (EC 2.7.1.69)3996"
[48] "Additional substrate-specific component CbiN of cobalt ECF transporter4043"
[49] "ATPase component CbiO of energizing module of cobalt ECF transporter4046"
[50] "ATPase component NikO of energizing module of nickel ECF transporter4047"
[51] "Ethanolamine operon regulatory protein4201"
[52] "Ethanolamine utilization polyhedral-body-like protein EutL4206"
[53] "Ethanolamine utilization polyhedral-body-like protein EutS4209"
[54] "Ethanolamine utilization protein EutG4211"
[55] "Ethanolamine utilization protein EutP4213"
[56] "Ethanolamine utilization protein EutQ4214"
[57] "Phosphate acetyltransferase (EC 2.3.1.8), ethanolamine utilization-specific4221"
[58] "Formiminoglutamase (EC 3.5.3.8)4276"
[59] "PTS system, mannitol-specific cryptic IIA component (EC 2.7.1.69)4324"
[60] "Uridine kinase family protein YggC homolog4327"
[61] "3-phenylpropionate dioxygenase, beta subunit (EC 1.14.12.19)4533"
[62] "Gene Transfer Agent (GTA) ORFG124768"
[63] "Gene Transfer Agent host specificity protein4770"
[64] "Glycerol dehydratase large subunit (EC 4.2.1.30)4989"
[65] "Glycerol dehydratase medium subunit (EC 4.2.1.30)4990"
[66] "Glycerol dehydratase small subunit (EC 4.2.1.30)4993"
[67] "Formiminoglutamase (EC 3.5.3.8)5396"
[68] "Ni,Fe-hydrogenase I cytochrome b subunit5495"
[69] "TonB-dependent heme receptor HutR5669"
[70] "Iron-sulfur cluster assembly scaffold protein NifU5710"
[71] "Predicted D-lactate dehydrogenase, Fe-S protein, FAD/FMN-containing5816"
[72] "Transcriptional repressor of the lac operon5861"
[73] "Transcriptional repressor of the lac operon5870"
[74] "UDP-galactose:(galactosyl) LPS alpha1,2-galactosyltransferase WaaW (EC 2.4.1.-)6234"
[75] "UDP-glucose:(glucosyl)lipopolysaccharide alpha-1,3-glucosyltransferase WaaO (EC 2.4.1.-)6236"
[76] "Outer membrane protein OmpK6378"
[77] "Malonate utilization transcriptional regulator6389"
[78] "PTS system, mannitol-specific cryptic IIA component (EC 2.7.1.69)6452"
[79] "Uncharacterized protein YidS6519"
[80] "Glutamine amidotransferase chain of NAD synthetase6962"
[81] "2,3-dihydroxy-2,3-dihydro-phenylpropionate dehydrogenase (EC 1.3.1.-)7029"
[82] "Oxaloacetate decarboxylase alpha chain (EC 4.1.1.3)7043"
[83] "[NiFe] hydrogenase nickel incorporation protein HybF7105"
[84] "Cytochrome c552 precursor (EC 1.7.2.2)7108"
[85] "Cytochrome c-type heme lyase subunit nrfE, nitrite reductase complex assembly7110"
[86] "Cytochrome c-type heme lyase subunit nrfF, nitrite reductase complex assembly7111"

[87] "Cytochrome c-type heme lyase subunit nrfG, nitrite reductase complex assembly7112"
[88] "Cytochrome c-type protein NrfB precursor7114"
[89] "Ferredoxin-type protein NapF (periplasmic nitrate reductase)7116"
[90] "Ferredoxin-type protein NapG (periplasmic nitrate reductase)7117"
[91] "NrfC protein7131"
[92] "NrfD protein7132"
[93] "Polyferredoxin NapH (periplasmic nitrate reductase)7136"
[94] "Iron-sulfur cluster assembly scaffold protein NifU7154"
[95] "Nitrogenase FeMo-cofactor scaffold and assembly protein NifE7164"
[96] "Sugar-1-epimerase YihR7303"
[97] "Copper sensory histidine kinase CusS7340"
[98] "Putative two-component response regulator and GGDEF family protein YeaJ7349"
[99] "Sensory histidine kinase AtoS7354"
[100] "Alpha-glucosides-binding periplasmic protein AglE precursor7550"
[101] "Periplasmic thiol:disulfide interchange protein, DsbA-like7558"
[102] "Phage minor tail protein7734"
[103] "2,3-dihydroxy-2,3-dihydro-phenylpropionate dehydrogenase (EC 1.3.1.-)7807"
[104] "2,3-dihydroxy-2,3-dihydro-phenylpropionate dehydrogenase (EC 1.3.1.-)7840"
[105] "3-phenylpropionate dioxygenase, beta subunit (EC 1.14.12.19)7842"
[106] "Phosphoglycerate transport regulatory protein PgtC7901"
[107] "Phosphoglycerate transport system sensor protein PgtB (EC 2.7.3.-)7902"
[108] "Phosphoglycerate transport system transcriptional regulatory protein PgtA7903"
[109] "PutR, transcriptional activator of PutA and PutP8274"
[110] "Propanediol dehydratase large subunit (EC 4.2.1.28)8288"
[111] "Propanediol dehydratase medium subunit (EC 4.2.1.28)8289"
[112] "Propanediol dehydratase reactivation factor large subunit8290"
[113] "Propanediol dehydratase small subunit (EC 4.2.1.28)8292"
[114] "Propanediol utilization polyhedral body protein PduT8299"
[115] "Propanediol utilization polyhedral body protein PduU8300"
[116] "Propanediol utilization protein PduL8301"
[117] "Propanediol utilization protein PduV8303"
[118] "Propionate kinase, propanediol utilization (EC 2.7.2.1)8305"
[119] "Putative iron-containing NADPH-dependent propanol dehydrogenase8306"
[120] "AMINOTRANSFERASE CLASS-I (EC 2.6.1.-)8332"
[121] "Protein YjgK, linked to biofilm formation8467"
[122] "Uncharacterized sugar kinase YeiI8537"
[123] "Oxaloacetate decarboxylase alpha chain (EC 4.1.1.3)8825"
[124] "Phage minor tail protein8940"
[125] "Alpha-D-GlcNAc alpha-1,2-L-rhamnosyltransferase (EC 2.4.1.-)9095"
[126] "TRAP-type transport system, large permease component, predicted N-acetylneuraminate transporter9841"
[127] "ABC-type Fe3+-siderophore transport system, ATPase component9895"
[128] "Ferric enterobactin uptake protein FepE9942"
[129] "Proofreading thioesterase in enterobactin biosynthesis EntH9948"
[130] "Cytochrome c55210060"
[131] "Cytochrome c552 precursor (EC 1.7.2.2)10062"

[132] "Sulfide dehydrogenase [flavocytochrome C] flavoprotein chain precursor (EC 1.8.2.-)10525"
[133] "Citrate lyase gamma chain, acyl carrier protein (EC 4.1.3.6)10584"
[134] "Uncharacterized membrane lipoprotein clustered with tellurite resistance proteins TehA/TehB10655"
[135] "Iron-regulated protein A precursor11059"
[136] "Additional substrate-specific component CbiN of cobalt ECF transporter11082"
[137] "ATPase component CbiO of energizing module of cobalt ECF transporter11084"
[138] "ATPase component NikO of energizing module of nickel ECF transporter11085"
[139] "Nickel responsive regulator NikR11092"
[140] "Nickel transport ATP-binding protein nikD2 (TC 3.A.1.5.3)11093"
[141] "Nickel transport ATP-binding protein nikE2 (TC 3.A.1.5.3)11095"
[142] "Nickel transport system permease protein nikB2 (TC 3.A.1.5.3)11098"
[143] "Nickel transport system permease protein nikC2 (TC 3.A.1.5.3)11100"
[144] "TcuR: regulates tcuABC genes used in utilization of tricarballylate11169"
[145] "Ammonia monooxygenase11170"
[146] "Periplasmic protein torT precursor11179"
[147] "Sensor protein torS (EC 2.7.3.-)11180"
[148] "tRNA-binding protein YgjH11245"
[149] "Putative two-component response regulator11543"
[150] "type I secretion system ATPase, LssB family (LapB)11709"
[151] "Probable electron transfer flavoprotein-quinone oxidoreductase YgcN (EC 1.5.5.-)11782"
[152] "Universal stress protein F11815"
[153] "Putative two-component response regulator and GGDEF family protein YeaJ11834"
[154] "Fimbriae usher protein StcC11839"
[155] "Uncharacterized sugar kinase YegV, PfkB family11847"
[156] "Uncharacterized transcriptional regulator YfaX, HTH-type12181"
[157] "Chorismate synthase (EC 4.2.3.5)12193"
[158] "Sensor protein of zinc sigma-54-dependent two-component system12323"
[159] "Proton/aspartate symport protein12349"
[160] "Proton/glutamate symport protein12350"

Lower abundance under turf:

[1] "Anthranilate dioxygenase reductase624"
[2] "FIG020413: transmembrane protein1691"
[3] "Lipid carrier protein IgrF2377"
[4] "2-keto-3-deoxy-D-arabino-heptulosonate-7-phosphate synthase I alpha (EC 2.5.1.54)2439"
[5] "2-keto-3-deoxy-D-arabino-heptulosonate-7-phosphate synthase I alpha (EC 2.5.1.54)3071"
[6] "Formate dehydrogenase related protein4591"
[7] "Cyclopropane-fatty-acyl-phospholipid synthase 2, CmaA2 (EC 2.1.1.79)6904"
[8] "METHOXY MYCOLIC ACID SYNTHASE 3 MMAA3 (METHYL MYCOLIC ACID SYNTHASE 3) (MMA3) (HYDROXY MYCOLIC ACID SYNTHASE)6910"
[9] "2-cys peroxiredoxin BAS1, chloroplast precursor8027"
[10] "decaprenyl diphosphate synthase8164"

[11] "undecaprenyl diphosphate synthase8166"
[12] "2',3'-cyclic-nucleotide 2'-phosphodiesterase (EC 3.1.4.16)8593"
[13] "2',3'-cyclic-nucleotide 2'-phosphodiesterase (EC 3.1.4.16)8739"

Higher abundance in ruderal:

[1] "2,3-butanediol dehydrogenase, R-alcohol forming, (R)- and (S)-acetoin-specific (EC 1.1.1.4)119"
[2] "2,3-butanediol dehydrogenase, S-alcohol forming, (S)-acetoin-specific (EC 1.1.1.76)121"
[3] "Ferredoxin-like protein174"
[4] "Ferredoxin-like protein FixX175"
[5] "Ferredoxin-like protein YdiT176"
[6] "outer membrane protein AlgE323"
[7] "Allantoate amidohydrolase (EC 3.5.3.9)369"
[8] "DNA-binding transcriptional activator of the allD operon373"
[9] "Formate dehydrogenase -O, gamma subunit (EC 1.2.1.2)471"
[10] "Thiosulfate reductase electron transport protein phsB485"
[11] "Thiosulfate reductase precursor (EC 1.-.-.-)486"
[12] "Arsenical resistance operon trans-acting repressor ArsD654"
[13] "Adenosylcobinamide-phosphate guanylyltransferase (EC 2.7.7.62)702"
[14] "Aliphatic sulfonate monooxygenase family, FMNH2- or F420-dependent757"
[15] "COG2833: uncharacterized protein780"
[16] "ATP-dependent RNA helicase Bcep18194_A5658850"
[17] "Ortho-halobenzoate 1,2-dioxygenase alpha-ISP protein OhbB1090"
[18] "Fructose-1,6-bisphosphatase, type V, archaeal (EC 3.1.3.11)1385"
[19] "Predicted signal-transduction protein containing cAMP-binding and CBS domains1422"
[20] "Carnitine operon protein CaiE1548"
[21] "Electron transfer flavoprotein, alpha subunit FixB1554"
[22] "Electron transfer flavoprotein, beta subunit FixA1555"
[23] "Transcriptional activatory protein CaiF1557"
[24] "Lycopene cyclase1575"
[25] "Phytoene desaturase, pro-zeta-carotene producing (EC 1.-.-.-)1584"
[26] "CRISPR-associated protein, CT1975 family1755"
[27] "Putative glycosyltransferase protein1823"
[28] "STRUCTURAL ELEMENTS; Cell Exterior; surface polysaccharides/antigens1825"
[29] "Replicative DNA helicase (EC 3.6.1.-) [SA14-24]1844"
[30] "Putative lipase in cluster with Phosphatidate cytidylyltransferase2013"
[31] "Predicted signal-transduction protein containing cAMP-binding and CBS domains2065"
[32] "FIG004655: Polysaccharide deacetylase2276"
[33] "Chlorophyllide reductase subunit BchY (EC 1.18.-.-)2353"
[34] "Chlorophyllide reductase subunit BchZ (EC 1.18.-.-)2354"
[35] "Geranylgeranyl hydrogenase BchP2356"
[36] "Light-independent protochlorophyllide reductase iron-sulfur ATP-binding protein ChlL (EC 1.18.-.-)2358"
[37] "Light-independent protochlorophyllide reductase subunit B (EC 1.18.-.-)2359"

[38] "Light-independent protochlorophyllide reductase subunit N (EC 1.18.-.-)2360"
[39] "Mg protoporphyrin IX monomethyl ester oxidative cyclase (aerobic) (EC 1.14.13.81)2361"
[40] "Protoporphyrin IX Mg-chelatase subunit H (EC 6.6.1.1)2366"
[41] "Glycine betaine transporter OpuD2395"
[42] "2,3-dihydroxy-2,3-dihydro-phenylpropionate dehydrogenase (EC 1.3.1.-)2467"
[43] "3-phenylpropionate dioxygenase, beta subunit (EC 1.14.12.19)2473"
[44] "Hca operon (3-phenylpropionic acid catabolism) transcriptional activator HcaR2479"
[45] "Mhp operon transcriptional activator2480"
[46] "TctA citrate transporter2485"
[47] "Aspartate aminotransferase family2534"
[48] "Adenosylcobinamide-phosphate guanylyltransferase (EC 2.7.7.62)2601"
[49] "Alpha-ribazole-5'-phosphate phosphatase (EC 3.1.3.73)2603"
[50] "Cobalamin synthase2606"
[51] "Threonine kinase in B12 biosynthesis2619"
[52] "Cation efflux system protein CusC precursor2622"
[53] "Additional substrate-specific component CbiN of cobalt ECF transporter2677"
[54] "Adenosylcobinamide-phosphate guanylyltransferase (EC 2.7.7.62)2679"
[55] "Alpha-ribazole-5'-phosphate phosphatase (EC 3.1.3.73)2683"
[56] "ATPase component CbiO of energizing module of cobalt ECF transporter2684"
[57] "Cobalamin synthase2692"
[58] "Substrate-specific component CbiM of cobalt ECF transporter2725"
[59] "Threonine kinase in B12 biosynthesis2728"
[60] "Transmembrane component CbiQ of energizing module of cobalt ECF transporter2729"
[61] "ABC transporter, permease protein YnjC2780"
[62] "COG0398: uncharacterized membrane protein2781"
[63] "Mercuric ion reductase (EC 1.16.1.1)2787"
[64] "Putative phosphatidylglycerophosphate synthase2790"
[65] "Geranylgeranyl hydrogenase BchP2891"
[66] "Hypothetical protein YbbP, contains nucleotide-binding domain of DisA bacterial checkpoint controller2933"
[67] "FIG002577: Putative lipoprotein precursor2967"
[68] "Colanic acid biosynthesis glycosyl transferase WcaE3038"
[69] "Colanic acid biosynthesis glycosyl transferase WcaL3039"
[70] "Colanic acid biosysnthesis glycosyl transferase WcaI3041"
[71] "GDP-mannose mannosyl hydrolase (EC 3.6.1.-)3046"
[72] "Lipopolysaccharide biosynthesis protein WzxC3047"
[73] "Multiple antibiotic resistance protein MarA3065"
[74] "Regulatory protein SoxS3066"
[75] "Lipoate synthase3101"
[76] "IncF plasmid conjugative transfer surface exclusion protein TraT3157"
[77] "Inner membrane protein YhaH3221"
[78] "Inner membrane protein YhaI3222"
[79] "CopG protein3286"
[80] "Copper resistance protein B3288"
[81] "Copper tolerance protein3293"

[82] "CRISPR-associated protein, CT1975 family3343"
[83] "Minor curlin subunit CsgB, nucleation component of curlin monomers3359"
[84] "D-allose ABC transporter, substrate-binding component3433"
[85] "DedA family inner membrane protein YdjZ3444"
[86] "Cytochrome cd1 nitrite reductase (EC:1.7.2.1)3471"
[87] "Nitric-oxide reductase subunit B (EC 1.7.99.7)3476"
[88] "Nitric-oxide reductase subunit C (EC 1.7.99.7)3477"
[89] "Nitrous-oxide reductase (EC 1.7.99.6)3481"
[90] "Nitrous oxide reductase maturation protein NosD3483"
[91] "Nitrous oxide reductase maturation transmembrane protein NosY3487"
[92] "Predicted nucleoside ABC transporter, permease 2 component3532"
[93] "Positive regulator of L-idonate catabolism3635"
[94] "Cytochrome cd1 nitrite reductase (EC:1.7.2.1)3661"
[95] "Heme d1 biosynthesis protein NirF3663"
[96] "Heme d1 biosynthesis protein NirJ3666"
[97] "Nitrite reductase associated c-type cytochorome NirN3668"
[98] "LysR family transcriptional regulator PA33983703"
[99] "LysR family transcriptional regulator YfiE3713"
[100] "Replicative DNA helicase (EC 3.6.1.-); intein-containing3900"
[101] "Replicative DNA helicase (EC 3.6.1.-) [SA14-24]3901"
[102] "Predicted nucleoside ABC transporter, permease 2 component3972"
[103] "Galactitol utilization operon repressor4004"
[104] "Additional substrate-specific component CbiN of cobalt ECF transporter4043"
[105] "ATPase component CbiO of energizing module of cobalt ECF transporter4046"
[106] "ATPase component NikO of energizing module of nickel ECF transporter4047"
[107] "ATPase component STY3232 of energizing module of queuosine-regulated ECF transporter4050"
[108] "ATPase component STY3233 of energizing module of queuosine-regulated ECF transporter4051"
[109] "Substrate-specific component CbiM of cobalt ECF transporter4063"
[110] "Substrate-specific component STY3230 of queuosine-regulated ECF transporter4074"
[111] "Substrate-specific component YkoE of thiamin-regulated ECF transporter for HydroxyMethylPyrimidine4079"
[112] "Transmembrane component CbiQ of energizing module of cobalt ECF transporter4082"
[113] "Transmembrane component STY3231 of energizing module of queuosine-regulated ECF transporter4092"
[114] "Ethanolamine utilization polyhedral-body-like protein EutK4205"
[115] "Ethanolamine utilization protein EutG4211"
[116] "Ethanolamine utilization protein EutP4213"
[117] "Ethanolamine utilization protein EutQ4214"
[118] "Phosphate acetyltransferase (EC 2.3.1.8), ethanolamine utilization-specific4221"
[119] "Acetoacetyl-CoA reductase (EC 1.1.1.36) of ethylmalonyl-CoA pathway4223"
[120] "Acetyl-CoA acetyltransferase (EC 2.3.1.9) of ethylmalonyl-CoA pathway4224"
[121] "Formiminoglutamase (EC 3.5.3.8)4276"
[122] "PTS system, mannitol-specific cryptic IIA component (EC 2.7.1.69)4324"
[123] "Uridine kinase family protein YggC homolog4327"

[124] "Flagellar basal-body P-ring formation protein FlgA4468"
[125] "3-phenylpropionate dioxygenase, beta subunit (EC 1.14.12.19)4533"
[126] "Formate dehydrogenase -O, gamma subunit (EC 1.2.1.2)4585"
[127] "Two-component sensor kinase YesM (EC 2.7.3.-)4655"
[128] "Fructose ABC transporter, substrate-binding component FrcB4674"
[129] "PTS system, fructose-specific IIB component (EC 2.7.1.69)4678"
[130] "Transcriptional regulator, GABA/putrescine utiliation cluster # 14721"
[131] "Gene Transfer Agent (GTA) ORFG124768"
[132] "Gene Transfer Agent host specificity protein4770"
[133] "Gene Transfer Agent portal protein4772"
[134] "Glycerol dehydratase medium subunit (EC 4.2.1.30)4990"
[135] "Glycerol dehydratase small subunit (EC 4.2.1.30)4993"
[136] "Fructose-1,6-bisphosphatase, type V, archaeal (EC 3.1.3.11)5149"
[137] "Cytochrome cd1 nitrite reductase (EC:1.7.2.1)5252"
[138] "Heme d1 biosynthesis protein NirJ5260"
[139] "Formiminoglutamase (EC 3.5.3.8)5396"
[140] "Hydantoinase5452"
[141] "Ni,Fe-hydrogenase I cytochrome b subunit5495"
[142] "Potassium uptake protein, integral membrane component, KtrA5512"
[143] "Predicted D-lactate dehydrogenase, Fe-S protein, FAD/FMN-containing5816"
[144] "Predicted L-lactate dehydrogenase, hypothetical protein subunit YkgG5821"
[145] "Transcriptional repressor of the lac operon5861"
[146] "Transcriptional repressor of the lac operon5870"
[147] "UPF0141 membrane protein YijP possibly required for phosphoethanolamine modification of lipopolysaccharide6043"
[148] "Lipoate synthase6046"
[149] "Lipopolysaccharide 1,2-N-acetylglucosaminetransferase (EC 2.4.1.56)6219"
[150] "UDP-galactose:(galactosyl) LPS alpha1,2-galactosyltransferase WaaW (EC 2.4.1.-)6234"
[151] "UDP-glucose:(glucosyl)lipopolysaccharide alpha-1,3-glucosyltransferase WaaO (EC 2.4.1.-)6236"
[152] "Rhamnulokinase RhaK in alpha-proteobacteria (EC 2.7.1.5)6274"
[153] "Outer membrane protein OmpK6378"
[154] "Malonate decarboxylase delta subunit6385"
[155] "Malonate utilization transcriptional regulator6389"
[156] "Inner membrane ABC transporter permease protein YcjO6402"
[157] "PTS system, mannitol-specific cryptic IIA component (EC 2.7.1.69)6452"
[158] "Transcriptional regulator of mannitol utilization, DeoR family protein6458"
[159] "GDP-mannose mannosyl hydrolase (EC 3.6.1.-)6469"
[160] "Uncharacterized protein YidS6519"
[161] "Mercuric ion reductase (EC 1.16.1.1)6541"
[162] "Mercuric ion reductase (EC 1.16.1.1)6543"
[163] "Propionate catabolism operon regulatory protein PrpR6755"
[164] "Molybdenum transport system protein ModD6812"
[165] "Multidrug efflux RND transporter MexD6848"
[166] "Multiple antibiotic resistance protein MarA6862"
[167] "Na(+) H(+) antiporter subunit A6866"

[168] "Na(+) H(+) antiporter subunit B6867"
[169] "Na(+) H(+) antiporter subunit C6868"
[170] "Na(+) H(+) antiporter subunit E6870"
[171] "Glutamine amidotransferase chain of NAD synthetase6962"
[172] "2,3-dihydroxy-2,3-dihydro-phenylpropionate dehydrogenase (EC 1.3.1.-)7029"
[173] "Oxaloacetate decarboxylase alpha chain (EC 4.1.1.3)7043"
[174] "Oxaloacetate decarboxylase beta chain (EC 4.1.1.3)7044"
[175] "Glycine betaine transporter OpuD7085"
[176] "[NiFe] hydrogenase nickel incorporation protein HybF7105"
[177] "Cytochrome c-type heme lyase subunit nrfE, nitrite reductase complex assembly7110"
[178] "Cytochrome c-type heme lyase subunit nrfF, nitrite reductase complex assembly7111"
[179] "Cytochrome c-type heme lyase subunit nrfG, nitrite reductase complex assembly7112"
[180] "Cytochrome c-type protein NrfB precursor7114"
[181] "Ferredoxin-type protein NapF (periplasmic nitrate reductase)7116"
[182] "Ferredoxin-type protein NapG (periplasmic nitrate reductase)7117"
[183] "NrfC protein7131"
[184] "NrfD protein7132"
[185] "Polyferredoxin NapH (periplasmic nitrate reductase)7136"
[186] "GDP-mannose mannosyl hydrolase (EC 3.6.1.-)7272"
[187] "Outer membrane sugar transport protein YshA7301"
[188] "Sugar-1-epimerase YihR7303"
[189] "Sugar kinase YihV7304"
[190] "Oxidase7306"
[191] "Putative two-component response regulator and GGDEF family protein YeaJ7349"
[192] "Sensory histidine kinase AtoS7354"
[193] "Outer membrane protein G precursor7383"
[194] "Regulatory protein SoxS7414"
[195] "L,D-transpeptidase YnhG7522"
[196] "Alpha-glucosides-binding periplasmic protein AglE precursor7550"
[197] "Periplasmic thiol:disulfide interchange protein, DsbA-like7558"
[198] "Phage head completion-stabilization protein7617"
[199] "Phage EaA protein7635"
[200] "Phage terminase, small subunit7691"
[201] "Phage tail assembly protein I7738"
[202] "2,3-dihydroxy-2,3-dihydro-phenylpropionate dehydrogenase (EC 1.3.1.-)7807"
[203] "2,3-dihydroxy-2,3-dihydro-phenylpropionate dehydrogenase (EC 1.3.1.-)7840"
[204] "3-phenylpropionate dioxygenase, beta subunit (EC 1.14.12.19)7842"
[205] "Phosphoglycerate transporter protein PgtP7900"
[206] "Phosphoglycerate transport regulatory protein PgtC7901"
[207] "Phosphoglycerate transport system sensor protein PgtB (EC 2.7.3.-)7902"
[208] "Photosynthetic reaction center M subunit7999"
[209] "Geranylgeranyl reductase (EC 1.3.1.83)8152"
[210] "Potassium uptake protein, integral membrane component, KtrA8201"
[211] "PutR, transcriptional activator of PutA and PutP8274"
[212] "Propanediol dehydratase large subunit (EC 4.2.1.28)8288"
[213] "Propanediol dehydratase small subunit (EC 4.2.1.28)8292"

[214] "Propanediol utilization polyhedral body protein PduB8295"
[215] "Propanediol utilization polyhedral body protein PduU8300"
[216] "Propanediol utilization protein PduL8301"
[217] "Propanediol utilization protein PduV8303"
[218] "Putative iron-containing NADPH-dependent propanol dehydrogenase8306"
[219] "Threonine kinase in B12 biosynthesis8307"
[220] "AMINOTRANSFERASE CLASS-I (EC 2.6.1.-)8332"
[221] "Arginine-tRNA-protein transferase (EC 2.3.2.8)8455"
[222] "Nondeblocking aminopeptidase YpdE (X-X-[^PR]- specific)8462"
[223] "PhaK-like protein8515"
[224] "Uncharacterized sugar kinase YeiI8537"
[225] "Uncharacterized lipoprotein YgeR precursor8657"
[226] "TldE/PmbA family protein, Beta/Gamma-proteobacterial subgroup8695"
[227] "Pyrimidine ABC transporter, transmembrane component 18774"
[228] "Oxaloacetate decarboxylase alpha chain (EC 4.1.1.3)8825"
[229] "Oxaloacetate decarboxylase beta chain (EC 4.1.1.3)8826"
[230] "Substrate-specific component STY3230 of queuosine-regulated ECF transporter8888"
[231] "Riboflavin transporter PnuX9130"
[232] "N-acylglucosamine 2-epimerase (EC 5.1.3.8)9822"
[233] "TRAP-type transport system, large permease component, predicted N-acetylneuraminate transporter9841"
[234] "ABC-type Fe3+-siderophore transport system, ATPase component9895"
[235] "Ferric enterobactin uptake protein FepE9942"
[236] "FIG005032: Putative cytoplasmic protein YbdZ in enterobactin biosynthesis operon9943"
[237] "Proofreading thioesterase in enterobactin biosynthesis EntH9948"
[238] "Serine phosphatase RsbU, regulator of sigma subunit10024"
[239] "Rhodanese domain protein UPF0176, Actinobacterial subgroup10040"
[240] "PTS system, fructose-specific IIB component (EC 2.7.1.69)10470"
[241] "Regulatory protein SoxS10523"
[242] "Cyclic beta-1,2-glucan modification transmembrane protein10559"
[243] "Uncharacterized membrane lipoprotein clustered with tellurite resistance proteins TehA/TehB10655"
[244] "Substrate-specific component YkoE of thiamin-regulated ECF transporter for HydroxyMethylPyrimidine10770"
[245] "Iron-regulated protein A precursor11059"
[246] "Molybdenum transport system protein ModD11078"
[247] "Additional substrate-specific component CbiN of cobalt ECF transporter11082"
[248] "ATPase component CbiO of energizing module of cobalt ECF transporter11084"
[249] "ATPase component NikO of energizing module of nickel ECF transporter11085"
[250] "Nickel responsive regulator NikR11092"
[251] "Nickel transport ATP-binding protein nikE2 (TC 3.A.1.5.3)11095"
[252] "Nickel transport system permease protein nikB2 (TC 3.A.1.5.3)11098"
[253] "Substrate-specific component CbiM of cobalt ECF transporter11105"
[254] "Transmembrane component CbiQ of energizing module of cobalt ECF transporter11107"
[255] "Putative lipase in cluster with Phosphatidate cytidylyltransferase11163"
[256] "TcuR: regulates tcuABC genes used in utilization of tricarballylate11169"

[257] "Sensor protein torS (EC 2.7.3.-)11180"
[258] "tRNA-binding protein YgjH11245"
[259] "tRNA-dependent lipid II--amino acid ligase11274"
[260] "type 1 fimbriae major subunit FimA11561"
[261] "Probable electron transfer flavoprotein-quinone oxidoreductase YgcN (EC 1.5.5.-)11782"
[262] "Universal stress protein F11815"
[263] "Putative HTH-type transcriptional regulator YdjF11830"
[264] "Putative oxidoreductase YdjL11831"
[265] "Putative two-component response regulator and GGDEF family protein YeaJ11834"
[266] "Fimbriae usher protein StcC11839"
[267] "Uncharacterized sugar kinase YegV, PfkB family11847"
[268] "Lipoate synthase12231"
[269] "TctA citrate transporter12263"
[270] "YjbF outer membrane lipoprotein12286"
[271] "Sensor protein of zinc sigma-54-dependent two-component system12323"
[272] "Zinc resistance-associated protein12324"
[273] "Proton/aspartate symport protein12349"
[274] "Proton/glutamate symport protein12350"

Lower abundance in ruderal:

[1] "Transcriptional activator protein solR207"
[2] "Transcription accessory protein (S1 RNA-binding domain)1782"
[3] "Transcription regulator in CO-DH cluster1950"
[4] "Alpha-1,4-N-acetylgalactosamine transferase PglH (EC 2.4.1.-)2110"
[5] "Transcription accessory protein (S1 RNA-binding domain)2152"
[6] "Transcription accessory protein (S1 RNA-binding domain)2256"
[7] "Lipid carrier protein IgrF2377"
[8] "2-keto-3-deoxy-D-arabino-heptulosonate-7-phosphate synthase I beta (EC 2.5.1.54)2440"
[9] "Periplasmic chorismate mutase I precursor (EC 5.4.99.5)2455"
[10] "Alpha-1,4-N-acetylgalactosamine transferase PglH (EC 2.4.1.-)2490"
[11] "Transcription regulator in CO-DH cluster2656"
[12] "2-keto-3-deoxy-D-arabino-heptulosonate-7-phosphate synthase I beta (EC 2.5.1.54)3072"
[13] "Ubiquinone biosynthesis monooxygenase UbiB4320"
[14] "Ferric vulnibactin receptor VuuA5653"
[15] "Predicted Lactate-responsive regulator, LysR family5818"
[16] "Cyclopropane-fatty-acyl-phospholipid synthase 2, CmaA2 (EC 2.1.1.79)6904"
[17] "Kynurenine formamidase (EC 3.5.1.9)6968"
[18] "NMN 5'-nucleotidase, extracellular (EC 3.1.3.5)6989"
[19] "1H-3-hydroxy-4-oxoquinaldine 2,4-dioxygenase7070"
[20] "Alpha-1,4-N-acetylgalactosamine transferase PglH (EC 2.4.1.-)7201"
[21] "Periplasmic chorismate mutase I precursor (EC 5.4.99.5)7802"
[22] "2-cys peroxiredoxin BAS1, chloroplast precursor8027"
[23] "decaprenyl diphosphate synthase8164"
[24] "undecaprenyl diphosphate synthase8166"
[25] "4-hydroxyproline epimerase (EC 5.1.1.8)8247"

[26] "N-3-oxohexanoyl-L-homoserine lactone synthase8916"
[27] "N-3-oxooctanoyl-L-homoserine lactone synthase8918"
[28] "Kynurenine formamidase (EC 3.5.1.9)11513"
[29] "Ubiquinone biosynthesis monooxygenase UbiB11753"
[30] "Phosphocarrier protein kinase/phosphorylase, nitrogen regulation associated12342"
[31] "Transcription accessory protein (S1 RNA-binding domain)12360"

Taxa at family level:

Higher abundance under turf:
[1] "Dongiaceae10" "Azospirillaceae14"
[3] "Geminicoccaceae21" "BIrii4135"
[5] "Sandaracinaceae40" "Geobacteraceae47"
[7] "Aeromonadaceae58" "TRA3-2063"
[9] "SC-I-8469" "Nitrosomonadaceae72"
[11] "Steroidobacteraceae80" "Rubinisphaeraceae101"
[13] "bacteriap25107" "Hymenobacteraceae113"
[15] "Flavobacteriaceae118" "Saprospiraceae130"
[17] "AKYH767134" "Rubritaleaceae157"
[19] "Verrucomicrobiaceae159" "Nitrospiraceae166"
[21] "Blastocatellaceae169" "Pyrinomonadaceae170"
[23] "Unknown_Family177" "Gaiellaceae179"
[25] "67-14181" "Iamiaceae184"
[27] "Microtrichaceae185" "Ilumatobacteraceae186"
[29] "Sporichthyaceae188" "Nakamurellaceae189"
[31] "Nocardioidaceae191" "Propionibacteriaceae192"
[33] "Geodermatophilaceae194" "Nocardiaceae196"
[35] "Streptomycetaceae199" "Pseudonocardiaceae200"
[37] "Micromonosporaceae203" "Micrococcaceae207"
[39] "Cellulomonadaceae211" "Intrasporangiaceae219"
[41] "Streptosporangiaceae223" "Clostridiaceae_1261"
[43] "AKYG1722270" "Roseiflexaceae273"
[45] "JG30-KF-CM45274" "Anaerolineaceae280"
[47] "Caldilineaceae281" "A4b282"
[49] "Saccharimonadaceae306" "Nitrososphaeraceae321"
[51] "Rhodobacteraceae347" "Rhizobiaceae348"
[53] "Methyloligellaceae349" "Hyphomicrobiaceae350"
[55] "Rhizobiales_Incertae_Sedis353" "Sphingomonadaceae354"
[57] "Devosiaceae355"
Lower abundance under turf:
[1] "Acetobacteraceae12" "Micropepsaceae17"
[3] "Polyangiaceae38" "Fimbriimonadaceae56"
[5] "Isosphaeraceae103" "Methylacidiphilaceae155"
[7] "Pedosphaeraceae156" "Acidobacteriaceae_(Subgroup_1)171"
[9] "Koribacteraceae172" "Solibacteraceae_(Subgroup_3)173"

[11] "Mycobacteriaceae195" "Actinospicaceae201"
[13] "Acidothermaceae221" "Chthonomonadaceae228"
[15] "Ktedonobacteraceae278" "Vermiphilaceae303"
[17] "0319-6G20326"

Higher abundance under ruderal:
[1] "Dongiaceae10" "Azospirillaceae14"
[3] "Geminicoccaceae21" "BIrii4135"
[5] "Sandaracinaceae40" "Pseudomonadaceae50"
[7] "Aeromonadaceae58" "TRA3-2063"
[9] "Burkholderiaceae66" "Xanthomonadaceae77"
[11] "Steroidobacteraceae80" "Rubinisphaeraceae101"
[13] "bacteriap25107" "Hymenobacteraceae113"
[15] "Cytophagaceae115" "Microscillaceae116"
[17] "Flavobacteriaceae118" "Saprospiraceae130"
[19] "AKYH767134" "Rubritaleaceae157"
[21] "Verrucomicrobiaceae159" "Nitrospiraceae166"
[23] "Blastocatellaceae169" "Unknown_Family177"
[25] "Gaiellaceae179" "67-14181"
[27] "Iamiaceae184" "Microtrichaceae185"
[29] "Ilumatobacteraceae186" "Sporichthyaceae188"
[31] "Nakamurellaceae189" "Nocardioidaceae191"
[33] "Propionibacteriaceae192" "Geodermatophilaceae194"
[35] "Nocardiaceae196" "Pseudonocardiaceae200"
[37] "Micromonosporaceae203" "Micrococcaceae207"
[39] "Cellulomonadaceae211" "Microbacteriaceae215"
[41] "Intrasporangiaceae219" "Peptococcaceae232"
[43] "Carnobacteriaceae244" "AKIW781268"
[45] "AKYG1722270" "Roseiflexaceae273"
[47] "JG30-KF-CM45274" "Anaerolineaceae280"
[49] "Caldilineaceae281" "A4b282"
[51] "Saccharimonadaceae306" "Nitrososphaeraceae321"
[53] "Rhodobacteraceae347" "Rhizobiaceae348"
[55] "Hyphomicrobiaceae350" "Rhizobiales_Incertae_Sedis353"
[57] "Sphingomonadaceae354" "Devosiaceae355"
Lower abundance under ruderal:
[1] "Xanthobacteraceae1" "Acetobacteraceae12"
[3] "Micropepsaceae17" "Polyangiaceae38"
[5] "Unknown_Family79" "Diplorickettsiaceae86"
[7] "CPla-3_termite_group89" "Gimesiaceae99"
[9] "Gemmataceae102" "Isosphaeraceae103"
[11] "Methylacidiphilaceae155" "Pedosphaeraceae156"
[13] "Xiphinematobacteraceae162" "Acidobacteriaceae_(Subgroup_1)171"
[15] "Koribacteraceae172" "Solibacteraceae_(Subgroup_3)173"
[17] "Mycobacteriaceae195" "Actinospicaceae201"
[19] "Acidothermaceae221" "Chthonomonadaceae228"

[21] "Ktedonobacteraceae278" "Vermiphilaceae303"
[23] "0319-6G20326"

Higher abundance under remnant:
None

Lower abundance under remnant:
None
