# Supplementary material for: Metagenomics Reveals Bacterial and Archaeal Adaptation to Urban Land-Use: N Catabolism, Methanogenesis, and Nutrient Acquisition
Source: Front Microbiol. 2019 Oct 10;10:2330. doi: 10.3389/fmicb.2019.02330 (PMC6795690; doi:10.3389/fmicb.2019.02330)
Supplement: Supplementary file 2 [file Table_1.pdf]

Supplemental figure S1:

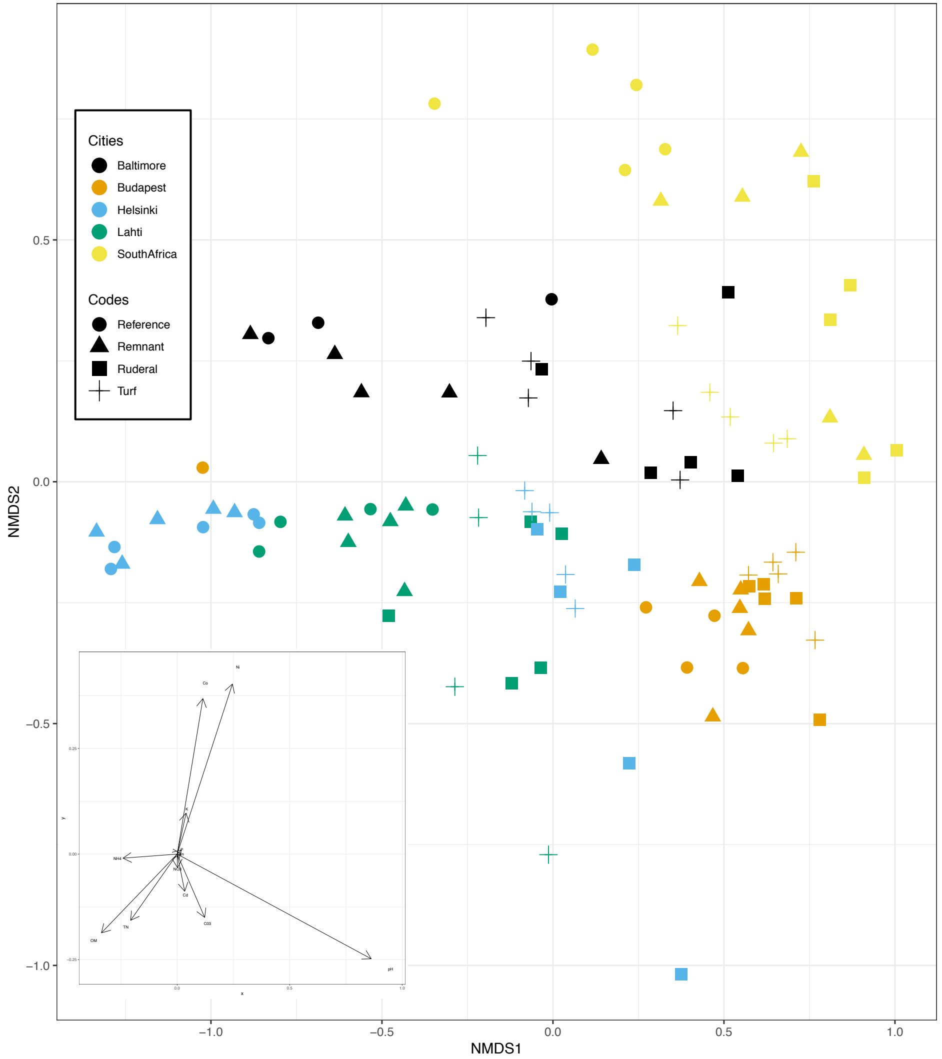

Figure S1: bacterial 16S amplicon community had significant city (PERMANOVA r^2=0.22, P=0.001) and land use effects (PERMANOVA r^2=0.09, P=0.001). There was also significant convergence in turf sites (ANOVA F = 3.57, P = 0.017), which is represented by the tighter clustering of turf in the ordination.

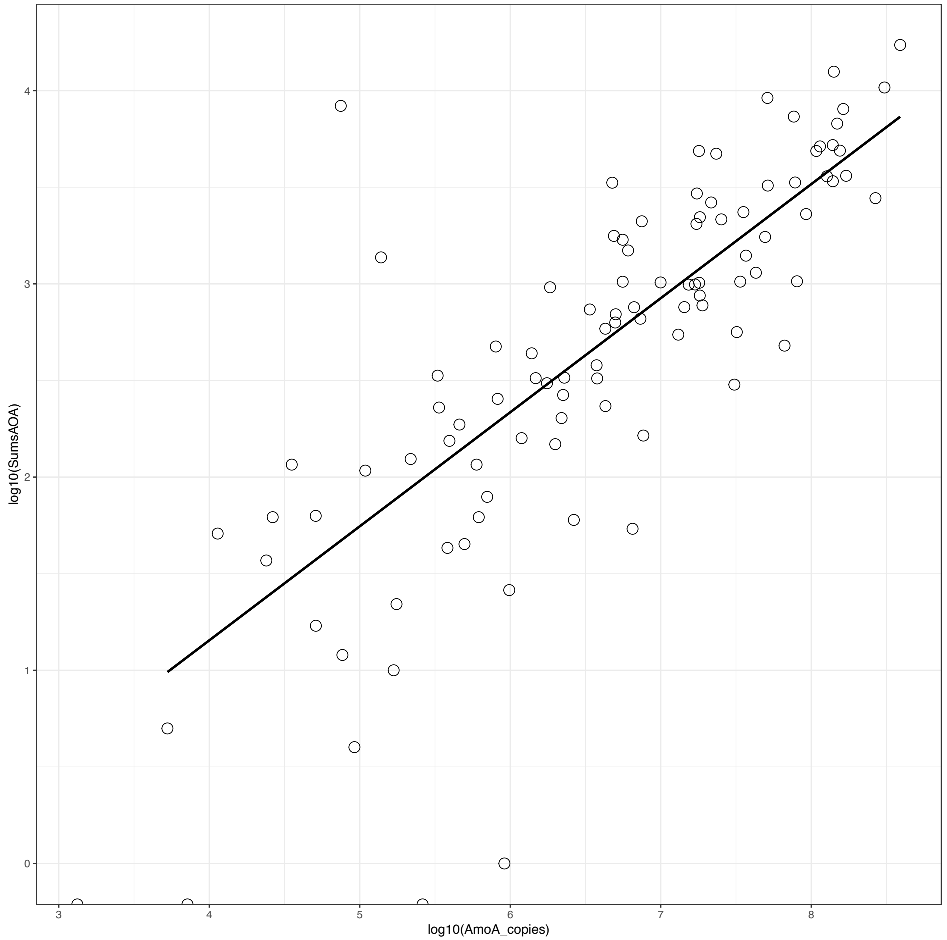

Figure S2: Correlation between log10 transformed QPCR and log10 transformed sequence counts shows high concordance between sequencing and quantitative estimation (Pearson r=0.78, P<0.001)

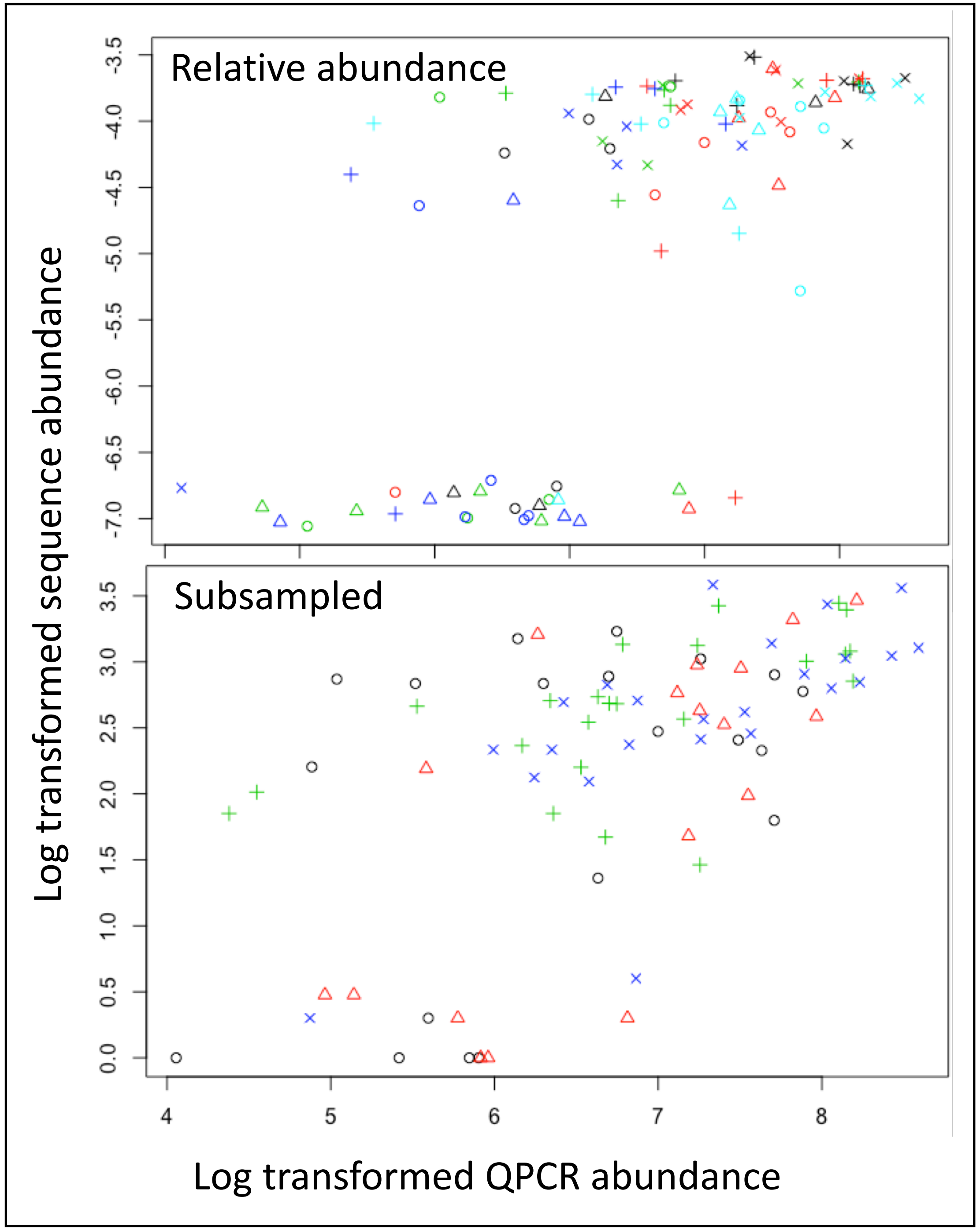

Figure S3: scatterplots of log10 transformed *amoA* abundance as estimated by QPCR versus shotgun sequenced. Two normalization methods were used; first we use relative abundance, then we account for density using a density-normalized subsampling routine. The latter approach reduces the effect of the artifact, but does not remove it entirely.

Methanogens: figure S4

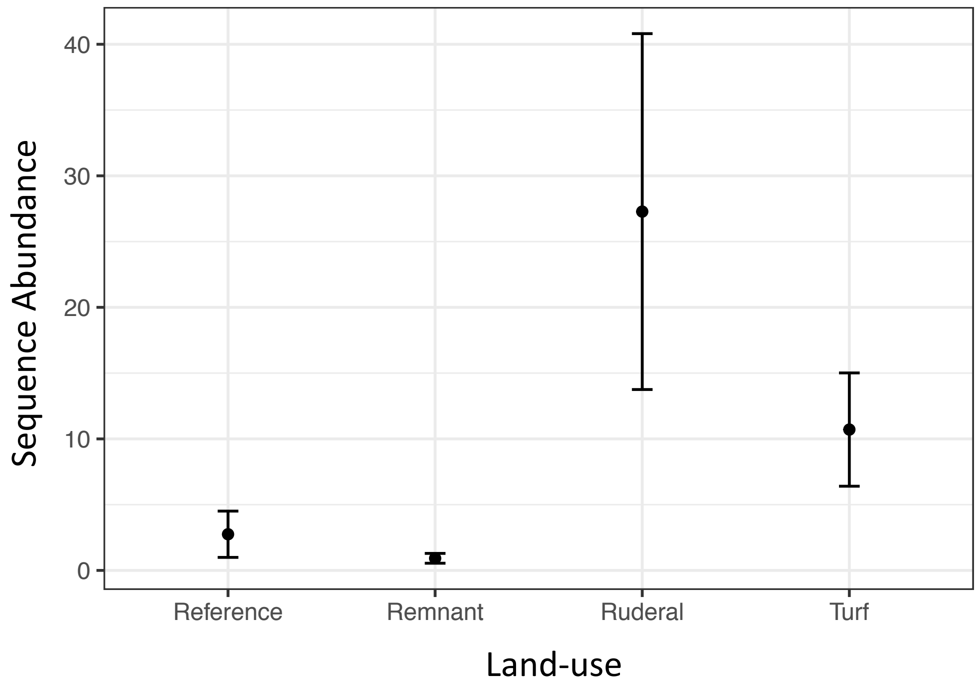

Figure S4: Abundance of putative archaeal methanogens under each land-use category. Ruderal sites have more methanogens than reference (adj. P=0.08) or remnant (adj. P=0.05) sites.

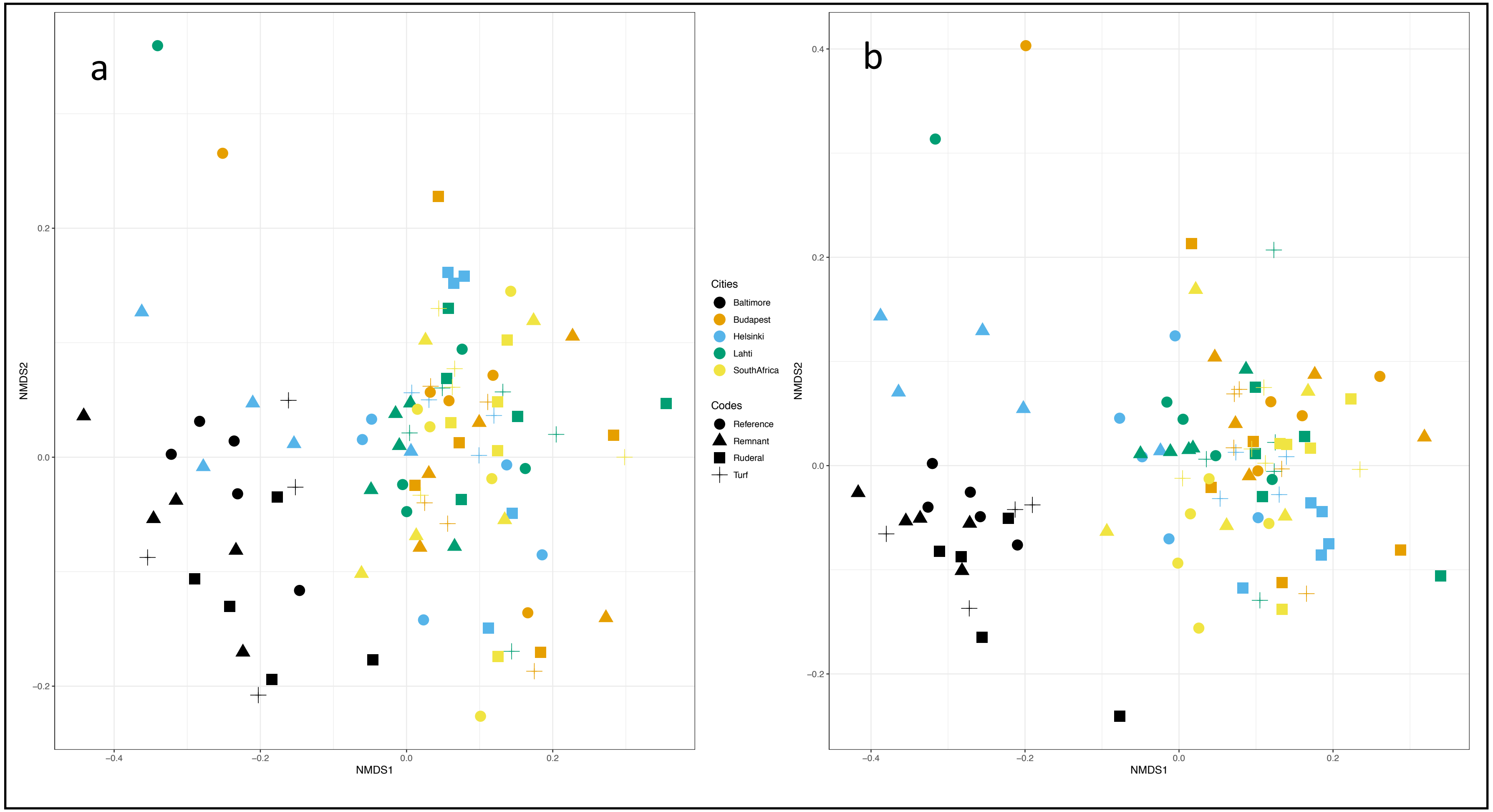

Figure S5: ordinations of functional profiles using Bray-Curtis similarity indexes and two different normalization methods: relative abundance (a) and standard-density subsampling (b). The latter results in stronger pattern differentiation and tighter correlations to environmental parameters (see Table S2)

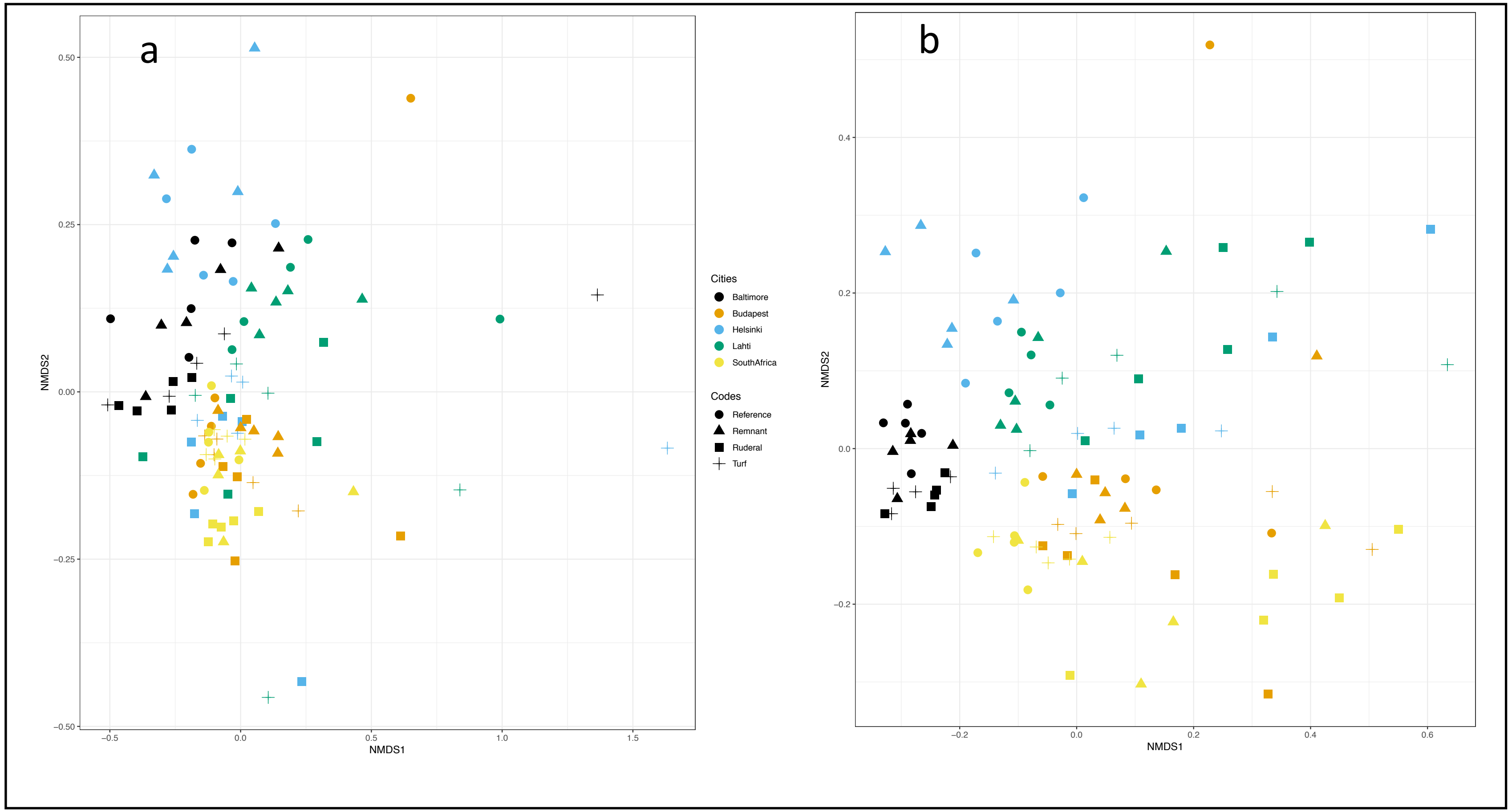

Figure S6: ordinations of mgrast annotated taxonomy profiles using Bray-Curtis similarity indexes and two different normalization methods: relative abundance (a) and standard-density subsampling (b). The latter results in stronger pattern differentiation and tighter correlations to environmental parameters (see Table S3)

Table S1: Summary table of total sequences submitted to MG-Rast; number of sequences assigned to known and unknown proteins and RNA in each sample; number of sequences removed for QC by the MG-Rast pipeline.

	Unknown Proteins	Total	Unknown	Known Rna	Known Protein	Failed QC
Final_001	7582168	22222133	21018497	25850415	1784663	5552616
Final_002	845195	2678640	31167146	22999512	1171014	38438077
Final_003	5145699	10096547	6759107	12839	2382295	1122829
Final_004	9108773	23217986	18323190	1564580	3614533	55829062
Final_005	39227530	11596432	48314528	23286131	23002363	6493708
Final_006	9476381	3095783	5343055	895630	11548152	5026683
Final_007	3806743	4727701	4706	2073302	1126912	402040
Final_008	14887550	9650519	4606903	5144759	23284931	1916642
Final_009	3350147	1598473	1006413	1009979	467589	2219675
Final_010	6003126	3668931	1998086	1506279	4812434	6003336
Final_011	568801	6037358	986449	1452342	6138901	389433
Final_012	14221633	9123345	5448406	10425554	1926363	8110019
Final_013	4654590	2647299	759297	2990035	4655014	2896913
Final_014	1992893	1673154	2118144	5495	3454192	3486618
Final_015	4301850	1288311	3962708	7279901	8620533	1975141
Final_016	5170027	10286	15863548	2478673	2746516	5458046
Final_017	4926730	332207	684605	1248347	5465511	4783980
Final_018	11250337	10986938	3478246	44895354	28646291	15209510
Final_019	5974685	9656783	2393797	5011060	2630486	5168625
Final_020	72980	241	58949	101438	23295	7781
Final_021	1793222	1457521	3819542	6065655	2048148	6009628
Final_022	2074126	5017726	1251761	4009820	3073365	3122566
Final_023	4915180	1717329	5871487	2822280	5449152	4060752
Final_024	576539	922262	2489631	1293808	277992	2363024
Final_025	246414	3887769	2894123	10189278	957256	2203716
Final_026	1769952	1595114	2606021	10211704	4822256	3705209
Final_027	1996369	652495	8605127	14813464	14354843	1832334
Final_028	514084	4367629	5949024	1465489	1831077	2301561
Final_029	172311	3725626	3883506	5494680	227721	5773440
Final_030	9136085	5821691	6448124	9056636	1748049	2620491
Final_031	2836484	3750467	2920659	9137380	2452927	4351803
Final_032	4498650	1282054	1339538	1606994	2484664	1747936
Final_033	17856	11553	12210	18100	38925	25600
Final_034	3456259	1863246	2913283	2903326	2287738	4795432
Final_035	3617246	2982138	279583	1028603	3455334	3331912
Final_036	2886493	5975654	7042616	1319225	452250	4656846
Final_037	802202	3491940	5117257	3923060	6451115	11141970
Final_038	137804	950167	1176005	243113	462062	2026985
Final_039	2781750	3929121	694702	2624768	3821311	3911840
Final_040	507894	849626	1860367	2073649	1804798	1231098
Final_041	1347272	426355	674721	1570607	2246257	780704
Final_042	4146228	2134875	2616892	6010425	3390066	5017254
Final_043	224829	83346	61313	52380	77062	183894
Final_044	2940882	3097151	4715291	131026	1948555	2554175
Final_045	84734	795298	1627071	801423	1575542	338400
Final_046	909326	588403	1059510	2878421	1135804	902592
Final_047	4545031	3100221	1997522	2638301	1075705	2451972
Final_048	1229376	2957258	1754121	2410992	466533	769376
Final_049	10206974	13441974	42583473	41431787	1728379	12647625
Final_050	8244745	2640791	7958589	3012773	1280644	3942134
Final_051	4119693	800743	2244168	2729666	5561461	36977
Final_052	2984381	1820639	3457902	789244	3128670	3638584
Final_053	631497	1331487	2498576	1651126	878190	1509412
Final_054	1996281	4497117	8190836	4440915	6527437	1886498
Final_055	137688	206210	225141	69796	523366	266287
Final_056	2676927	1010674	1104519	2743215	2646525	2421620
Final_057	1129394	1414370	3224424	1461781	774912	739019
Final_058	245211	459110	843861	873284	468848	637138
Final_059	1335116	592330	988612	233603	3186044	886399
Final_060	1362461	2670329	1394529	2154483	2558353	1103441
Final_061	1375538	12087643	7039898	3029707	9745239	1251687
Final_062	309659	103451	456612	110093	317723	264566
Final_063	5626029	7752251	3493573	715811	5525447	2670905
Final_064	7884937	3201610	3662729	663465	5444946	2580545
Final_065	4765400	2309758	2914396	3374681	2196513	1891928
Final_066	2669035	4519233	3497658	7117355	2466290	8066409
Final_067	3904387	3055631	4508772	2817448	1538344	3101010
Final_068	703175	1279591	6535608	1724329	1584847	1243666
Final_069	2674526	392306	1196712	4204207	1522619	2819474
Final_070	540302	255266	219866	180007	519999	114128
Final_071	1578418	3084210	686160	1709937	7655954	6504125
Final_072	5141696	2990661	4813668	3557238	4748790	715755
Final_073	1526477	2886931	395203	1416620	624896	955489
Final_074	3715431	5947343	4669799	2997629	1935175	6806071
Final_075	735221	9756662	9011382	4897145	5269392	6246122
Final_076	1460960	4736278	5119426	6282024	2428518	5609494
Final_077	1502513	927063	4953477	1945999	1925296	3771300
Final_078	3592956	382680	2781808	1934062	735242	4901856
Final_079	3134390	672045	2263780	1762474	1956613	473434
Final_080	2744979	2134465	1149025	1584501	645179	738739
Final_081	2373059	3169427	2149639	5591535	3442504	2741368
Final_082	3451070	328338	2333596	4660306	357792	1733438
Final_083	3187846	2777495	3525900	4576045	4404012	2513682
Final_084	6993983	4551013	2369830	3189124	3407620	6760850
Final_085	1047619	8942134	2398500	1185276	6376903	5471604
Final_086	2041751	1863167	336193	3481811	3237409	1967445
Final_087	9866017	3768276	1403117	4977382	5932642	1583442
Final_088	806370	333288	1018353	528303	1095877	247077
Final_089	2384814	6902171	2601001	614537	7055017	877688
Final_090	2161478	8170187	2525694	985268	5465077	2512156
Final_091	3646698	4271554	6105441	4734704	518019	4170188
Final_092	1030230	2004465	1515727	1793058	6605470	261990
Final_093	4183069	600800	3222436	3247215	2000160	3248856
Final_094	2972335	5010082	6748398	4610638	4022943	6075568
Final_095	2615925	3254960	6590525	1980223	417947	8699288
Final_096	1785336	12018157	3340112	4470046	1649750	4742727
Final_097	5404977	2597944	373964	2946460	5075852	3647671
Final_098	1710336	2263274	5547585	2424402	4237587	3858232
Final_099	342158	2076079	1560398	3495308	8446922	972979
Final_100	464177	4689737	1267719	2185712	1808394	3748433

Table S2: Side-by-side significance tables of environmental correlations for function data. Change in value columns are the density normalized values subtracted from the relative abundance values. Therefore a negative value in r^2 represents an increase in goodness of fit by density normalization; and a non-negative p value represents a greater significance by density normalization. Values highlighted in green represent instances where density normalization increased the goodness of fit or decreased the p-value (more significant) of environmental covariates when density normalization was applied.

	Density Normalized		Relative Abundance		Change in value	
	r^2	Pr(>r)	r^2	Pr(>r)	r^2	Pr(>r)
pH	0.1597	0.003	0.157	0.002	-0.0027	-0.001
C_org	0.1385	0.002	0.0697	0.041	-0.0688	0.039
CaCO3	0.0487	0.104	0.0461	0.12	-0.0026	0.016
K2O	0.1489	0.002	0.1282	0.003	-0.0207	0.001
P2O5	0.0887	0.032	0.0541	0.111	-0.0346	0.079
NH4	0.0585	0.074	0.0459	0.131	-0.0126	0.057
NO3	0.1507	0.002	0.141	0.002	-0.0097	0
TotalN	0.1763	0.001	0.0675	0.049	-0.1088	0.048
Total Cd	0.0128	0.536	0.0353	0.202	0.0225	-0.334
Total Co	0.0074	0.719	0.0031	0.885	-0.0043	0.166
Total Ni	0.0463	0.123	0.0526	0.105	0.0063	-0.018
Totan Zn	0.0279	0.297	0.0076	0.705	-0.0203	0.408

Table S3: Side-by-side significance tables of environmental correlations for metagenomic taxonomy data. Change in value columns are the density normalized values subtracted from the relative abundance values. Therefore a negative value in r^2 represents an increase in goodness of fit by density normalization; and a non-negative p value represents a greater significance by density normalization. Values highlighted in green represent instances where density normalization increased the goodness of fit or decreased the p-value (more significant) of environmental covariates when density normalization was applied.

	Density Normalized		Relative Abundance		Change in value	
	r^2	Pr(>r)	r^2	Pr(>r)	r^2	Pr(>r)
pH	0.6062	0.001	0.6071	0.001	0.0009	0
C_org	0.4179	0.001	0.3889	0.001	-0.029	0
CaCO3	0.0896	0.02	0.0733	0.044	-0.0163	0.024
K2O	0.0278	0.295	0.0015	0.932	-0.0263	0.637
P2O5	0.0695	0.036	0.0523	0.096	-0.0172	0.06
NH4	0.2098	0.001	0.2461	0.002	0.0363	0.001
NO3	0.0574	0.059	0.0217	0.388	-0.0357	0.329
TotalN	0.3354	0.001	0.3182	0.002	-0.0172	0.001
Total Cd	0.0017	0.93	0	0.998	-0.0017	0.068
Total Co	0.1339	0.003	0.0353	0.213	-0.0986	0.21
Total Ni	0.2	0.001	0.1059	0.015	-0.0941	0.014
Totan Zn	0.0088	0.685	0.037	0.161	0.0282	-0.524
